# Cadmium, arsenic, and molybdenum removal from water using a tantalum MOF

**DOI:** 10.1186/s11671-026-04963-2

**Published:** 2026-09-29

**Authors:** Vahidreza Amiresmaeili, S. Morteza Mousavi, Ghasem Sargazi, Shabnam Sadri-Moghaddam

**Affiliations:** 1 Water Resources Department, Civil Engineering Faculty, K. N. Toosi University of Technology, Tehran, Iran; 2 Noncommunicable Diseases Research Center, Bam University of Medical Sciences, Bam, Iran; 3 Environment Department, Civil Engineering Faculty, K. N. Toosi University of Technology, Tehran, Iran

**Keywords:** Ta-MOF, Heavy metal removal, Adsorption, Response surface methodology, Water treatment

## Abstract

Access to clean water is increasingly threatened by rapid industrialization, population growth, and the discharge of heavy metals into aquatic systems, posing serious risks to human health and ecological balance. The development of advanced materials with high adsorption efficiency and multifunctionality is therefore essential for sustainable water treatment. In this study, a novel tantalum-based metal–organic framework (Ta-MOF) was synthesized via conventional and ultrasound-assisted methods and evaluated for the removal of cadmium (Cd), arsenic (As), and molybdenum (Mo) from aqueous solutions. The ultrasound-assisted route produced materials with improved crystallinity, particle dispersion, and structural uniformity, as confirmed by FTIR, SEM, EDS, XRD, and BET analyses. The adsorption process was systematically optimized using Response Surface Methodology (RSM), considering key operational parameters including pH, initial metal concentration, adsorbent dosage, and contact time. Maximum removal efficiencies of 89.76% (As), 94.32% (Mo), and 88.94% (Cd) were achieved under optimized conditions, with pH values around neutral and contact times of 39.77–46.25 min. Kinetic studies followed the pseudo-second-order model, indicating that the adsorption rate is strongly dependent on active site availability and suggesting a surface interaction-controlled process. Isotherm analyses showed good agreement with the Langmuir model, indicating monolayer adsorption on a relatively homogeneous surface. In addition, Ta-MOF exhibited moderate antibacterial activity against Escherichia coli and Staphylococcus aureus. Overall, the findings highlight the potential of Ta-MOF as a dual-functional material for heavy metal removal and microbial control, offering a promising approach for integrated water treatment in contaminated environments.

**Figure:**
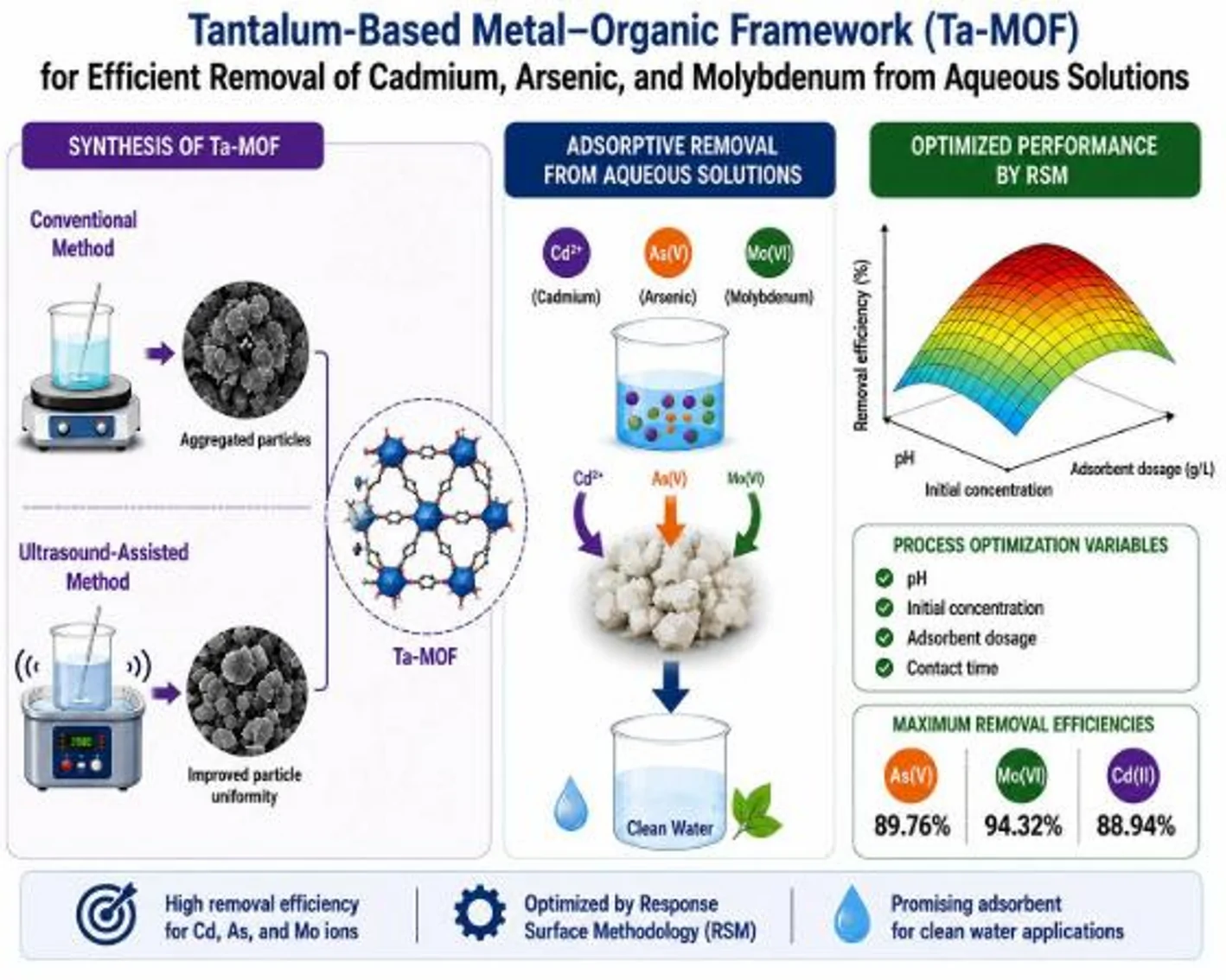

## Introduction

Environmental degradation is recognized as one of the most pressing global challenges, with water pollution being particularly urgent due to accelerated industrialization and urban expansion [1–3]. The 2024 UNESCO report highlights the severity of this crisis, noting that approximately 2.2 billion people lack access to safely managed drinking water, and 3.5 billion remain without safely managed sanitation [4]. Climate change is expected to exacerbate water scarcity, potentially affecting nearly half of the global population, while the United Nations projects a 40% shortfall in water resources within the next 15 years. Water pollution also threatens aquatic ecosystems, with UNESCO reporting the annual death of roughly one million marine organisms due to contamination [5, 6]. Partially treating water before injection and allowing natural attenuation during percolation helps mitigate these effects [7].

Water pollution arises from a variety of organic and inorganic contaminants, primarily from industrial activities, with heavy metal ions posing the most serious threat due to their toxicity and non-biodegradable nature [8, 9]. Arsenic (As), molybdenum (Mo), and cadmium (Cd) are particularly hazardous, as even trace levels can cause severe environmental and health impacts [10, 11]. Their high densities and atomic masses, combined with release from industrial wastewater, agricultural runoff, and domestic sources, underscore the urgent need for effective removal strategies [12–14].

The mentioned pollutants enter the human body through various exposure routes, including the ingestion of contaminated food and water, as well as inhalation of polluted air. These metals persist in the body because they cannot be broken down naturally, resulting in accumulation that poses serious health threats. For instance, prolonged exposure to arsenic has been associated with several cancers, while cadmium exposure is linked to damage in renal function and bone health [15]. While molybdenum is essential in small quantities for specific metabolic functions, overexposure may cause adverse health effects, such as liver damage and joint problems [16–18].

To mitigate the toxic impacts of heavy metals, numerous treatment approaches have been developed for their removal from contaminated water. Conventional methods encompass chemical precipitation [19], ion exchange [20, 21], and adsorption [22] utilizing materials such as activated carbon and mineral-based adsorbents. Among these, adsorption stands out as a preferred technique because of its affordability, operational simplicity, and limited generation of hazardous waste. Nonetheless, traditional adsorbents frequently exhibit poor selectivity and slow adsorption rates, which restrict their efficiency in selectively removing specific metals such as arsenic, molybdenum, and cadmium.

Optimizing operational parameters in the adsorption process is essential to maximize the removal efficiency of heavy metals from aqueous solutions. Although adsorption is inherently effective, its performance can be substantially enhanced by adjusting key variables. For example, the solution pH affects the ionization states of both the adsorbent surface and metal ions, thereby influencing their interactions. Likewise, the initial concentration of metal ions determines the concentration gradient, which drives mass transfer and impacts adsorption capacity. The quantity of adsorbent used is vital for providing adequate surface area for metal binding, while the contact time dictates the duration of interaction between adsorbent and ions, affecting the overall adsorption extent. By systematically optimizing these parameters, it is possible to achieve maximal removal efficiency, reduce operational expenses, and minimize environmental harm by lowering the concentration of residual metals in treated water [23–25].

Over the last decade, metal-organic frameworks (MOFs) have gained significant attention as precisely engineered porous materials with high surface areas and tunable structures, showing strong potential for removing heavy metals such as arsenic (As), molybdenum (Mo), and cadmium (Cd) from water [26, 27]. Also known as porous coordination polymers (PCPs), MOFs are crystalline frameworks formed by the coordination of metal ions with organic ligands [28], combining the advantageous properties of both inorganic and organic components [29]. Functionalized MOFs exhibit strong affinity for metal ions, enhancing their adsorption performance. Various metal ions including Zn(II), Cu(II), Mg(II), Ca(II), Ln(III), Al(III), Zr(IV), Ti(III), Co(II), and Cd(II) allow for diverse geometries such as tetrahedral, octahedral, and square planar structures [30]. The inherent structural flexibility of MOFs, along with their tunable porosity, structural flexibility, and chemical versatility, makes them particularly well-suited for environmental applications, notably in the adsorption and elimination of heavy metal ions from polluted water sources.

Despite these advantages, the application of MOFs in real water treatment systems still faces important limitations. Although many MOF-based adsorbents show high removal efficiencies for heavy metal ions under controlled conditions, their performance often decreases in complex aqueous environments due to ion competition, changes in ionic strength, and the presence of natural organic matter. In addition, issues such as water stability, limited selectivity in multi-component systems, and insufficient validation under real wastewater conditions remain major challenges. Recent studies have focused on improving these limitations through structural design, functional group modification, and selection of more stable metal centers, which has led to notable progress in adsorption performance and material durability [31–34].

The primary aim of this study was to evaluate the adsorption performance of a synthesized tantalum-based metal-organic framework (Ta-MOF) for the removal of heavy metal ions from aqueous solutions. Tantalum was selected because of its excellent chemical stability, corrosion resistance, and strong metal–ligand coordination behavior, which contribute to the formation of efficient framework structures suitable for adsorption applications. The Ta-MOF synthesized using 2,6-pyridine dicarboxylic acid as the organic linker was chosen due to its structural stability and the presence of active sites that can facilitate interactions with heavy metal ions. To optimize the adsorption process, Response Surface Methodology (RSM) was employed to systematically investigate the effects of operational parameters and their interactions, enabling the identification of optimal adsorption conditions. To the best of our knowledge, this is among the first studies to investigate the application of a Ta-MOF for the removal of arsenic, cadmium, and molybdenum from aqueous media. Furthermore, the effects of CON an ULTRA methods were systematically compared to assess their influence on material properties and adsorption performance. The combination of a tantalum-based MOF, comparative synthesis approaches, and statistical process optimization distinguishes the present work from previously reported studies and highlights its potential for environmental remediation applications.

## Materials and methods

Tantalum (V) chloride (molecular weight: 358.21 g·mol^− 1^, purity: 99.4%) and 2,6-pyridine dicarboxylic acid (molecular weight: 167.12 g·mol^− 1^, purity: 99.3%) were acquired from Sigma-Aldrich. Cadmium nitrate (Cd(NO_3_)_2_·4H_2_O), sodium arsenate (Na_2_HAsO_4_·7H_2_O), and ammonium molybdate ((NH_4_)_6_Mo_7_O_24_·4H_2_O) were used to prepare the stock solutions of Cd^2+^, As^5+^, and Mo^6+^. All solutions were prepared using double-distilled water. The materials utilized in this study were employed as received without undergoing any additional purification steps. XRD patterns were recorded in reflection mode using a powder X-ray diffractometer with copper Kα radiation (λ = 0.15406 nm), spanning a 2θ range of 10° to 0° with a step width of 0.01°. Scanning electron microscopy (SEM) was carried out using a MIRA3TESCAN-XMU to observe the morphological features. Energy dispersive spectroscopy (EDS) with a MIRA3-XMU EDS detector was used for elemental analysis. For SEM, the samples were coated with gold. FTIR spectra were recorded as potassium bromide (KBr) pellets on a SHIMADZU FT/IR-8400 spectrometer, covering the wavenumber range of 500 to 4000 cm⁻¹. Nitrogen adsorption–desorption isotherms were measured at 77 K (Belsorp mini II). The specific surface area was calculated using the Brunauer–Emmett–Teller (BET) method, and pore size distribution was obtained using the BJH method. Prior to these measurements, each sample (0.03 g) was heated to 180 °C under vacuum for 2 h.

### Synthesis of MOF

In this study, two different synthesis approaches were used to prepare the target compound. For the conventional synthesis (CON), 0.1791 g (0.5 mmol) of tantalum chloride and 0.0835 g (0.5 mmol) of 2,6-pyridine dicarboxylic acid were dissolved in 32 mL of double-distilled water. A brief ultrasonic treatment of 5 min was applied only to facilitate dissolution and obtain a homogeneous precursor solution. The mixture was then stirred magnetically for 6 h at room temperature to complete the synthesis process.

**Fig. 1:**
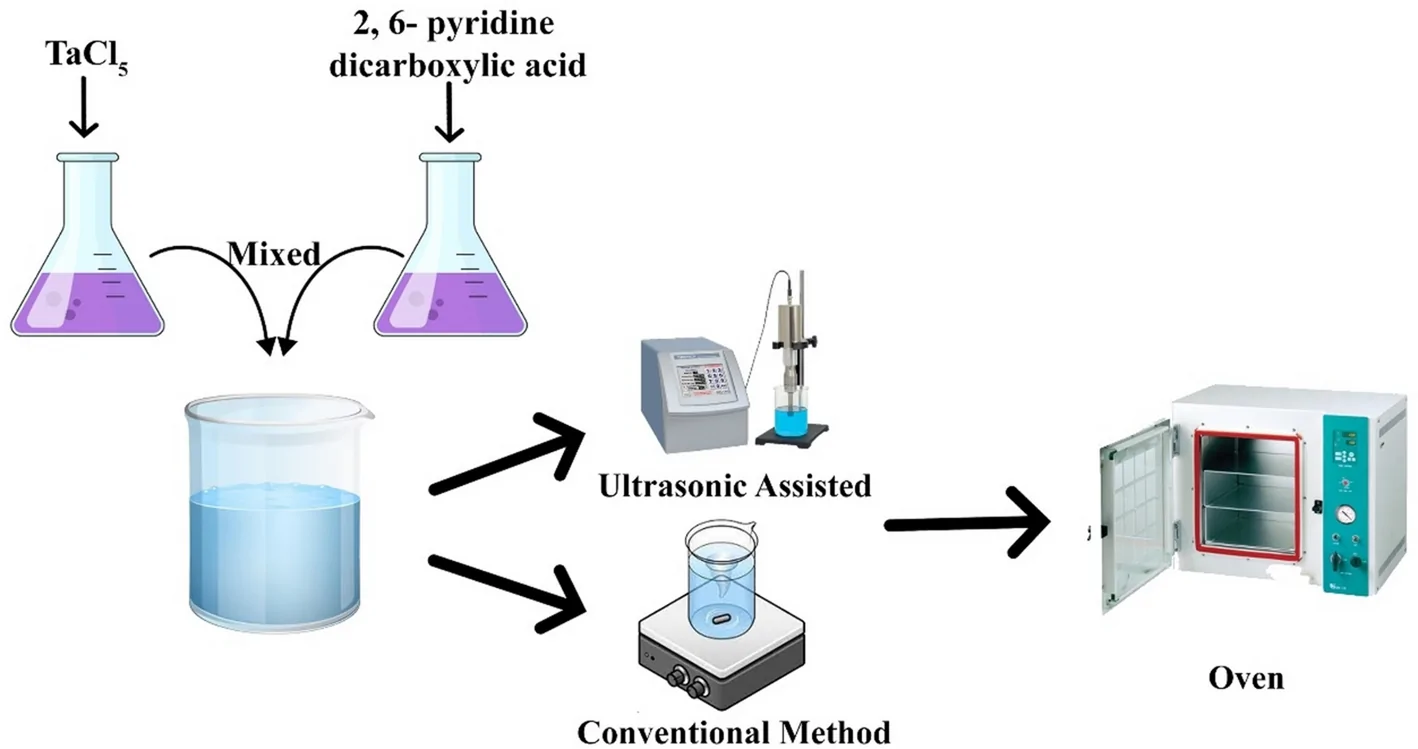
Schematic representation of the synthesis steps of Ta-MOF

For the ultrasound-assisted synthesis (ULTRA), the same precursor solution was prepared and transferred into a Pyrex tube [35]. The solution was exposed to ultrasonic irradiation at a frequency of 20 kHz and a power of 290 W for 30 min, where ultrasound served as the primary driving force for synthesis. The resulting products from both methods were collected by centrifugation, washed three times with water, and finally dried in a vacuum oven. The synthesis procedures are illustrated in Fig. 1.

### Adsorption test

The batch adsorption technique was employed to systematically evaluate the efficiency of the adsorption process in eliminating heavy metal ions from aqueous solutions. Standard stock solutions of As, Mo, and Cd ions, each prepared to a concentration of 1000 ppm through a dilution method using deionized water, served as the experimental media. Within each stock solution, a defined amount of tantalum (V) MOF was introduced into 25 mL flasks to ensure uniform distribution. These flasks were subsequently agitated on a shaker at 150 rpm for 2 h, maintaining a stable room temperature throughout the process. The pH levels of the solutions were meticulously adjusted using 0.1 N sodium hydroxide (NaOH) and nitric acid (HNO_3_) to establish controlled experimental conditions. Following this, spectral analysis was performed to measure the concentration of As, Mo, and Cd ions using atomic absorption (AA) spectrometry, providing precise quantification of the pollutants. After conducting the experiments, both kinetic and isotherm analyses were carried out to evaluate the adsorption characteristics. The adsorption capacity (q_t_), expressed in mg·g⁻¹, and the efficiency of removal %R were calculated using standard equations, yielding valuable insights into the dynamics of the adsorption process. Moreover, adsorption equilibrium was achieved over an extended period, where the adsorbate concentration on the adsorbent (q_e_) and the concentration in solution (C_e_) stabilized, indicating the completion of the adsorption process.

1 $${\rm{Removal}}\, {\rm{Efficiency}} (\%)\:=\frac{({C}_{i}-{C}_{e})}{{C}_{i}}\times\:100$$

To ensure the reliability and reproducibility of the experimental results, all adsorption trials were performed in triplicate. For each set of measurements, the half-range error was calculated as a statistical measure to reflect the variability between the maximum and minimum values observed across the replicates. This approach allowed for a more accurate estimation of experimental uncertainty, providing confidence in the consistency and precision of the adsorption data. The incorporation of half-range error analysis thus reinforced the validity of the experimental design and supported the robustness of the conclusions drawn from the adsorption performance.

### Experimental design

To optimize the adsorption removal process, an experimental design based on response surface methodology was employed using Design Expert Software. Key variables, including pH, initial concentration, adsorbent dosage, and contact time, were systematically adjusted to maximize the pollutant removal efficiency. Table 1 presents the variables and their respective ranges used in the optimization process.

**Table 1:** Key variables for experimental design

Factor	Name	Units	Minimum	Maximum
A	pH	-	3.00	9.00
B	Initial Concentration	ppm	10.00	100.00
C	Adsorbent Amount	mg	10.00	50.00
D	Time	min	5.00	60.00

### Kinetic study

The experiments investigating adsorption kinetics involved the use of a desired amount of the tantalum (V) MOF adsorbent with varying initial concentrations of pollutants over different time intervals ranging from 5 to 120 min in separate trials. After centrifugation to separate the liquid phase from the solid sorbent, the residual concentrations were determined using atomic absorption spectrometry. The amount of pollutants adsorbed at any specific time (q_t_), expressed in mg·g⁻¹, was calculated according to the following equation:

2 $$\:{\mathrm{q}}_{\mathrm{t}}=\frac{\left({\mathrm{C}}_{\mathrm{f}}-{\mathrm{C}}_{\mathrm{i}}\right)\mathrm{V}}{\mathrm{m}}$$

The first-order and second-order kinetic models used in this study are given in Eqs. (3) and (4).

3 $$\:\mathrm{ln}\left({\mathrm{q}}_{\mathrm{e}}-{\mathrm{q}}_{\mathrm{t}}\right)=\mathrm{l}\mathrm{n}\:{\mathrm{q}}_{\mathrm{e}}-\frac{{\mathrm{k}}_{1}\mathrm{t}}{2.303}$$

4 $$\:\frac{\mathrm{t}}{{\mathrm{q}}_{\mathrm{t}}}=\frac{1}{{\mathrm{k}}_{2}{{\mathrm{q}}_{\mathrm{e}}}^{2}}+\frac{\mathrm{t}}{{\mathrm{q}}_{\mathrm{e}}}$$

In these equations, q_t_ and q_e_ (mg·g⁻¹) denote the adsorption capacities of the components on the Ta-MOF at a specific time t (min) and at equilibrium, respectively. The constants k_1_ (min^− 1^) and k_2_ (g·mg^-1^.min^− 1^) represent the rate constants for the pseudo-first-order and pseudo-second-order models, respectively.

### Adsorption isotherms

Different adsorption isotherms were employed to investigate the adsorption behavior of heavy metal ions on the tantalum (V) MOF and to understand their interaction with the adsorbent surface. A range of initial metal ion concentrations (10 to 100 ppm) was used, while maintaining a constant adsorbent dosage in each experiment. After reaching equilibrium, the mixtures were centrifuged to separate the solid adsorbent from the solution, and the residual metal ion concentrations were quantified using atomic absorption spectrophotometry.

The adsorption data were analyzed using Langmuir (Eq. 5), Freundlich (Eq. 6), and Temkin (Eq. 7) isotherm models. Their linearized forms are expressed as follows:

5 $$\:\frac{{\mathrm{C}}_{\mathrm{e}}}{{\mathrm{q}}_{\mathrm{e}}}=\frac{{\mathrm{C}}_{\mathrm{e}}}{{\mathrm{q}}_{\mathrm{M}\mathrm{a}\mathrm{x}}}+\frac{1}{{\mathrm{q}}_{\mathrm{M}\mathrm{a}\mathrm{x}}{\mathrm{K}}_{\mathrm{L}}}$$

6 $$\:\mathrm{ln}{\mathrm{q}}_{\mathrm{e}}={\frac{1}{\mathrm{n}}}_{\mathrm{F}}\mathrm{ln}{\mathrm{C}}_{\mathrm{e}}+\mathrm{ln}{\mathrm{K}}_{\mathrm{F}}$$

7 $$\:{\mathrm{q}}_{\mathrm{e}}={{\upbeta\:}}_{\mathrm{T}}\mathrm{ln}({\mathrm{C}}_{\mathrm{e}})+{{\upbeta\:}}_{\mathrm{T}}\mathrm{ln}({\mathrm{K}}_{\mathrm{T}})$$

Here, Ce (mg**·**L⁻¹) represents the equilibrium concentration of metal ions in solution, while qmax (mg.g⁻¹) denotes the maximum monolayer adsorption capacity. βT is defined as RT/bT, where T (K) is the absolute temperature, R is the universal gas constant (8.314 J**·**mol⁻¹**·**K⁻¹), and bT (J**·**mol⁻¹) is the Temkin isotherm constant. The calculated isotherm parameters are discussed in detail in the Results and Discussion section.

### Assessment of antibacterial properties

The antibacterial performance of the synthesized Ta-MOF was investigated against two reference bacterial strains: Staphylococcus aureus (PTCC 1112) and Escherichia coli (PTCC 1330). The evaluation was carried out using a standard agar diffusion technique on Mueller-Hinton agar (Merck, Germany) as the growth medium. Bacterial cultures were grown for 18 to 24 h and subsequently adjusted to a turbidity equivalent to 0.5 McFarland standard to ensure consistent inoculum density. Sterile Petri plates were filled with 20 mL of Mueller-Hinton agar and uniformly inoculated with the prepared bacterial suspensions. Using aseptic techniques, wells were punched into the solidified agar surface, and 50 µL of Ta-MOF solution (50 mg·mL^− 1^ in dimethyl sulfoxide, DMSO) was introduced into each well. Plates were incubated at 37 °C for 24 to 48 h. Antibacterial activity was quantified by measuring the clear zones formed around each well, indicating inhibition of bacterial growth. DMSO served as the solvent control to confirm it had no intrinsic antimicrobial effect. All experiments were performed in triplicate to ensure consistency and reliability of results.

## Results and discussion

To comprehensively investigate the structural integrity of the synthesized compound, two distinct methodologies, namely conventional synthesis (designated as CON) and ultrasonic-assisted synthesis (designated as ULTRA), were employed. A characterization study was conducted on both samples to evaluate their respective properties and structural attributes. This examination is critical, as it enables a comparative analysis of the effects that each synthesis technique has on the material’s structural characteristics.

### Characterization

The characterization process typically involves various analytical techniques such as XRD, SEM, EDS, BET, and FTIR, which provide valuable insights into the crystallinity, morphology, and elemental composition of the samples. By applying these methods, the study aims to elucidate how the different synthesis approaches influence not only the structural integrity but also the overall performance of the synthesized compound in potential applications. The comparison of the results obtained from both synthesis techniques will facilitate a deeper understanding of the relationship between the synthesis method and the resultant material properties. This knowledge is crucial for optimizing synthesis protocols in order to enhance the performance and applicability of the compound in various fields, including catalysis, adsorption, and environmental remediation. Ultimately, the findings of this characterization study will contribute to the ongoing development of more effective and versatile materials for a wide range of applications.

#### FTIR spectroscopy analysis

**Fig. 2:**
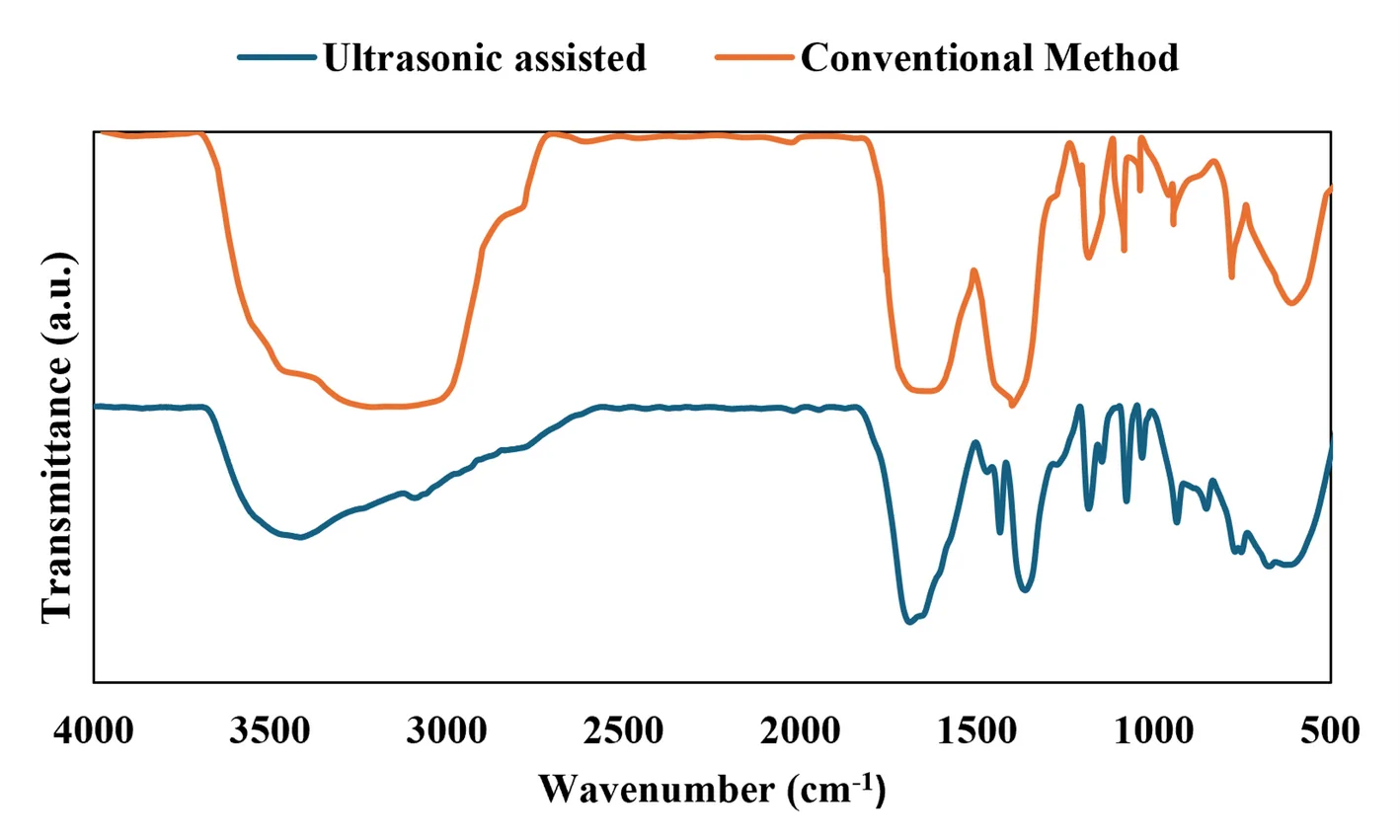
FTIR spectra of the synthesized MOF CON and ULTRA Methods

Fourier Transform Infrared (FTIR) spectroscopy was used to analyze the functional groups present in the Ta-MOF synthesized via CON and ULTRA methods (Fig. 2). The spectra of both samples exhibit similar characteristic absorption bands, confirming the presence of the same organic linker and general framework chemistry. The main differences are observed in peak intensity and slight shifts, which can be associated with variations in crystallinity and local coordination environments resulting from the different synthesis routes [36]. Both samples show absorption bands in the low wavenumber region (600–800 cm^-1^), which are associated with metal–oxygen vibrations within the framework [37]. Bands observed in the range of 750–800 cm^-1^ and around 900 cm^-1^ are attributed to aromatic C–H bending and C–N stretching vibrations from the pyridine-based linker. The regions around 1400 cm⁻¹ and 1600–1700 cm^-1^ correspond to carboxylate functional groups of the 2,6-pyridine dicarboxylic acid linker. Broad absorption bands in the 3200–3400 cm^-1^ range are related to hydroxyl groups and adsorbed water molecules within the structure [37, 38]. An additional weak band observed around 2600 cm^-1^ in the conventionally synthesized sample may be associated with hydrogen-bonding interactions or residual adsorbed species. Overall, the FTIR results confirm successful incorporation of the organic linker into the Ta-MOF framework for both synthesis methods. The observed differences in peak intensity and sharpness suggest variations in structural order and local bonding environment, which are consistent with differences in crystallinity between the two samples [39]. 

#### Morphological Analysis using scanning electron microscopy (SEM)

**Fig. 3:**
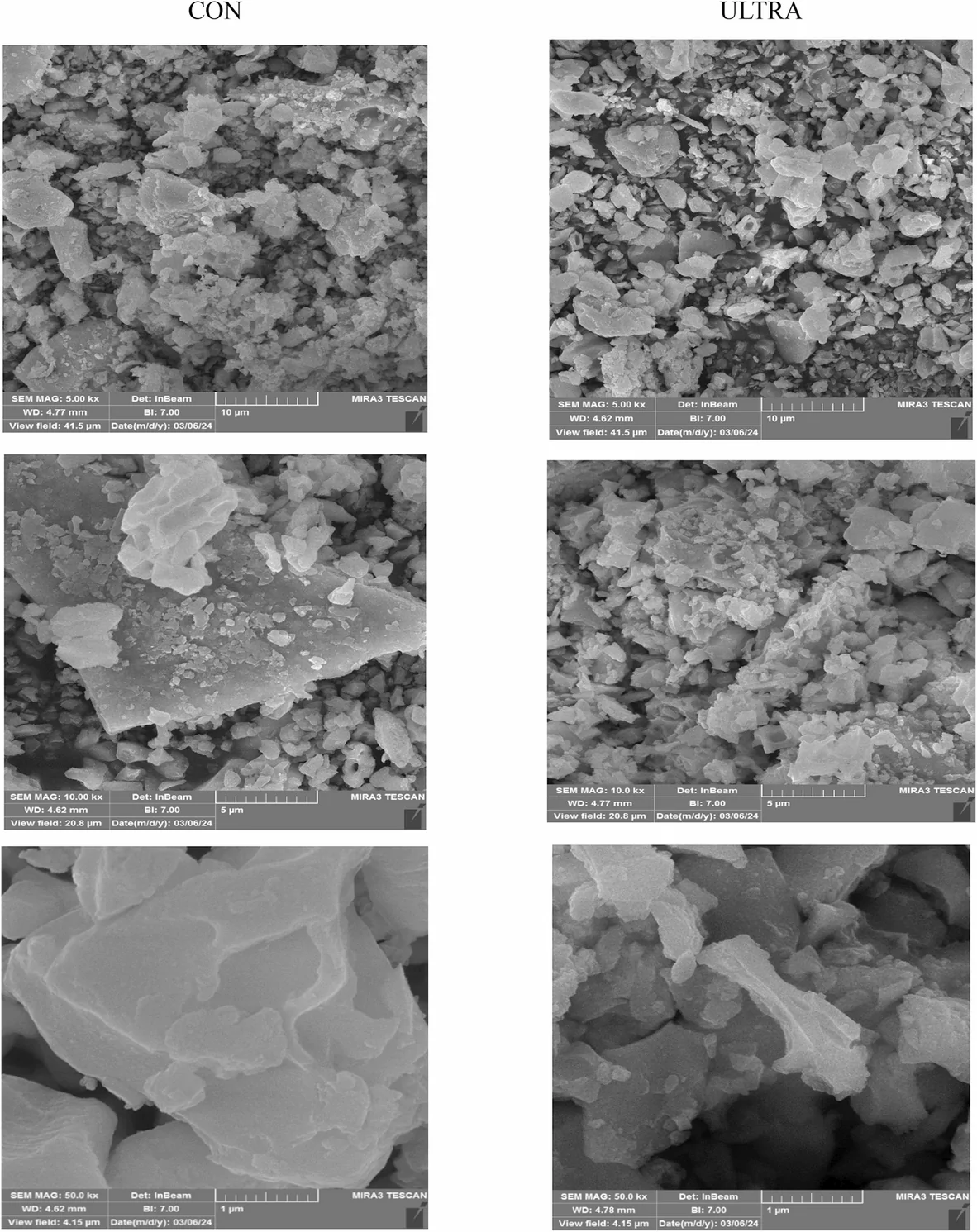
SEM images of the samples prepared by CON and ULTRA methods at different magnifications

The SEM images of the Ta-MOF samples synthesized by both conventional and ultrasonic-assisted methods are presented in Fig. 3. The SEM images demonstrate a clear dependence of Ta-MOF morphology on the synthesis route, where the CON method results in a compact and highly aggregated microstructure, whereas the ULTRA route produces a more dispersed and structurally open architecture. In the conventionally synthesized sample, the SEM micrographs at 5, 10, and 50 Kx reveal densely packed clusters composed of irregular plate-like and granular particles with poorly defined boundaries. This morphology reflects an uncontrolled nucleation and growth process, where particle coalescence dominates over discrete crystallite formation. As a result, the structure becomes compact with limited interparticle voids, rough surface features, and reduced accessible surface area, which can negatively affect adsorption and catalytic performance.

In contrast, as shown in Fig. 3, the ultrasonic-assisted Ta-MOF exhibits a noticeably improved morphological profile characterized by reduced agglomeration and better particle dispersion. The particles appear more fragmented and loosely packed, forming aggregates with clearer interparticle voids across all magnification levels. This indicates that ultrasonic irradiation enhances nucleation efficiency while restricting excessive particle growth and fusion, leading to a more controlled development of the microstructure. Consequently, the ULTRA sample presents a more open architecture with improved surface accessibility and more favorable textural characteristics for mass transfer processes. 

A direct comparison between the two synthesis methods confirms that ultrasonic treatment plays a significant role in regulating crystal growth behavior, shifting the system from dense aggregated clusters toward a more dispersed and accessible morphology. This structural improvement is particularly relevant for functional applications, as reduced agglomeration and enhanced particle uniformity increase the availability of active surface sites and facilitate diffusion within the MOF framework. 

Overall, the SEM results collectively confirm that ULTRA method yields a more structurally favorable Ta-MOF compared to the CON method, with reduced aggregation, improved dispersion, and enhanced microstructural accessibility, all of which contribute to its superior potential for adsorption and catalytic applications. 

**Fig. 4:**
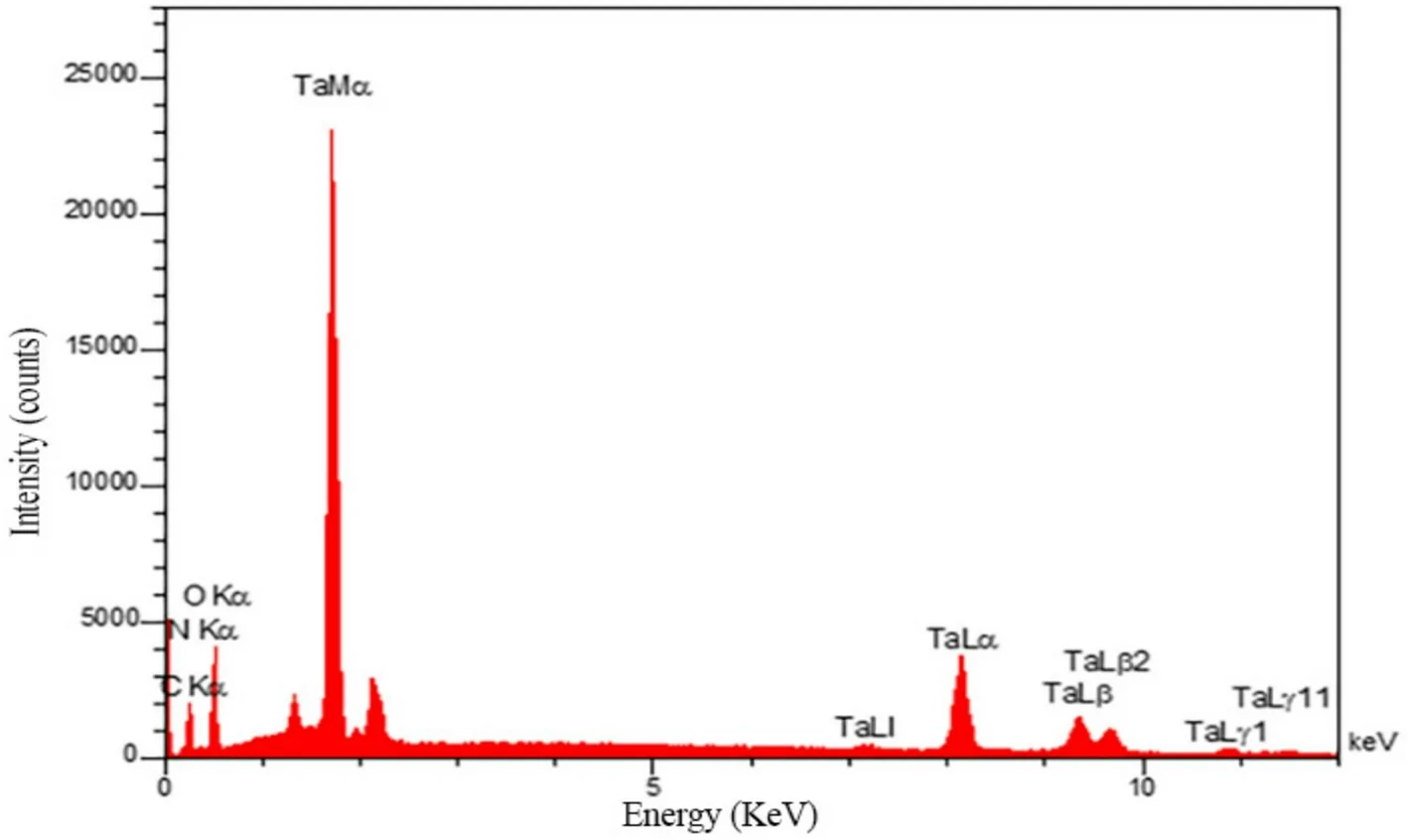
EDS of the sample prepared by ULTRA method

The EDS spectrum (Fig. 4) confirms the presence of Ta, C, N, and O, consistent with the incorporation of tantalum and the 2,6-pyridinedicarboxylic acid (ligand). The characteristic Ta and ligand-derived signals were observed, with no additional impurity elements detected within the EDS detection limit, supporting the formation of the Ta-containing coordination material.

#### X-ray diffraction (XRD) patterns

**Fig. 5:**
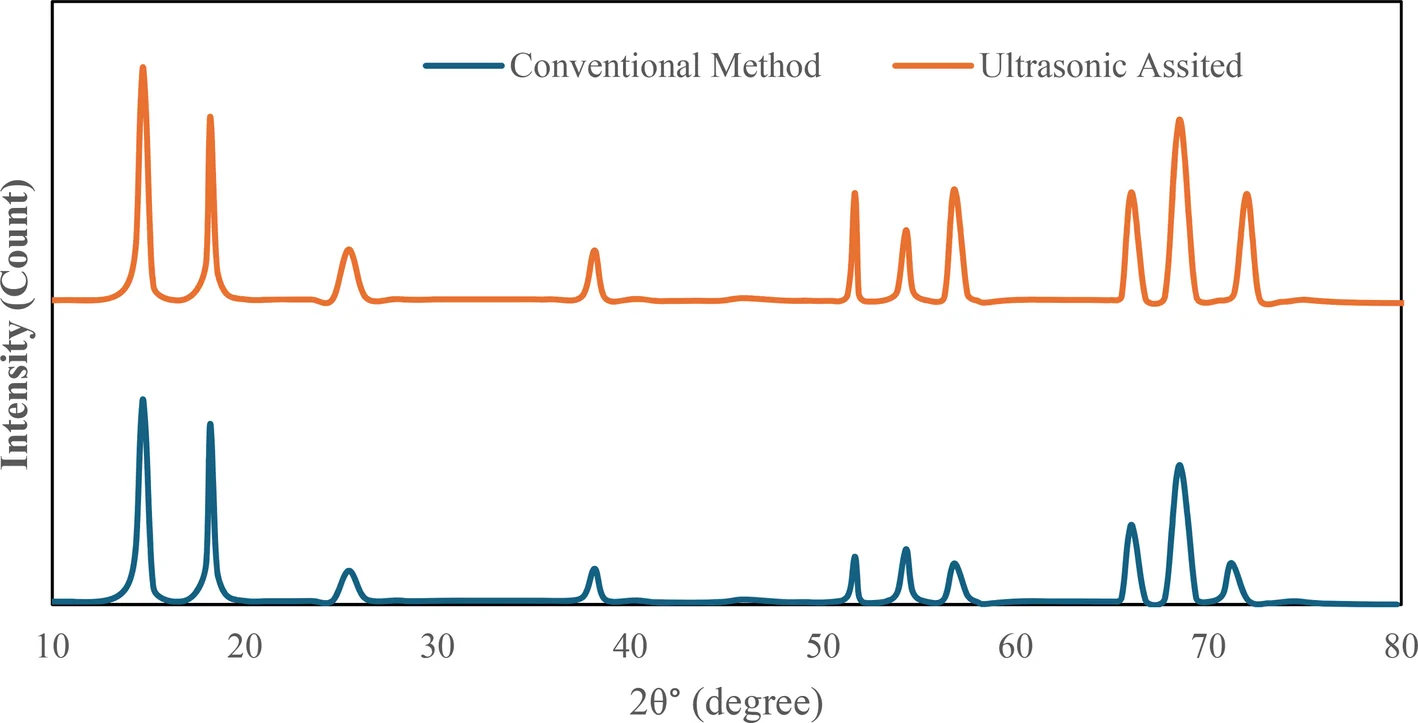
XRD patterns of Ta-MOF synthesized by CON and ULTRA methods

The X-ray diffraction (XRD) patterns of the tantalum-based MOF (Ta-MOF) were thoroughly examined to reveal critical details regarding its crystallographic structure (shown in Fig. 5). The XRD pattern displayed distinct diffraction peaks at various 2θ angles, corresponding to specific crystallographic planes. At a 2θ angle of 14.9°, the diffraction peak was assigned to the (011) plane, indicating a larger interlayer spacing. The peak at 18.7° was indexed to the (040) plane, indicating another distinct crystallographic orientation, highlighting the structural diversity within the material. A peak at 25.8° was assigned to the (120) plane, confirming the complexity of the atomic arrangement, while the peak at 39.6° was associated with the (051) plane, signifying a more compact atomic arrangement due to the higher diffraction angle. Peaks at 51.9° and 54.1° corresponded to the (032) and (071) planes, further reflecting the intricate atomic layering within the triclinic structure [35].

The peak at 56.9° was indexed to the (081) plane, followed by peaks at 66.0° and 68.4°, assigned to the (181) and (0100) planes, respectively, revealing a more densely packed crystal lattice. Finally, the peak at 71.6° corresponded to the (073) plane, one of the most tightly packed atomic layers in the Ta-MOF structure, underscoring the material’s high crystallinity. The broadening of these diffraction peaks, analyzed using the Scherrer equation, indicated nanoscale crystallite sizes of the Ta-MOF particles, suggesting the potential for unique material properties such as enhanced reactivity and modified electronic characteristics. Comparing the Ta-MOF synthesized via ULTRA methods with that produced by CON synthesis revealed differences in their XRD patterns. The ULTRA sample exhibited sharp, well-defined peaks at the same 2θ positions as the CON sample but with significantly higher intensities, reflecting improved crystallinity and structural purity. Notably, the peak intensities in the 50°- 60° range for the ULTRA sample were nearly double those of the CON sample, indicating a more ordered atomic structure. Additionally, the peak at 71.6° in the ULTRA sample shifted to 70° in the CON sample, accompanied by a reduction in intensity, suggesting the presence of structural defects and impurities. The superior crystallinity of the Ta-MOF synthesized using ULTRA methods can be attributed to the enhanced nucleation and crystal growth promoted by ultrasonic energy, resulting in a more uniform crystal structure. In contrast, the CON synthesis produced a sample with lower crystallinity, which can be attributed to less controlled nucleation and crystal growth processes. The XRD results indicate that ULTRA promotes a more efficient crystal formation pathway and improved structural ordering, resulting in a higher degree of crystallinity in the Ta-MOF. This enhanced crystallinity reflects a more well-developed and stable framework structure, which is expected to improve the accessibility and effectiveness of active sites, thereby supporting stronger surface interactions relevant to metal adsorption applications.

#### BET technique

The Brunauer–Emmett–Teller (BET) and Langmuir methods were employed to evaluate the surface areas of the synthesized Ta-MOF samples, as these characteristics directly influence the accessibility of active sites and interactions with metal ions. According to Table 2, the CON sample exhibited a higher BET surface area (2.73 m²·g⁻¹) and Langmuir surface area (65.33 m²·g⁻¹) compared to the ULTRA sample (1.40 m²·g⁻¹ and 32.72 m²·g⁻¹, respectively). The total pore volumes of CON and ULTRA were similar (0.006 and 0.005 cm³·g⁻¹), while the average pore diameter of ULTRA (14.70 nm) was larger than that of CON (8.63 nm). These results indicate that while the CON synthesis yields a material with slightly greater accessible surface area, adsorption behavior is also influenced by surface chemistry and active site availability rather than surface area alone.

**Table 2:** BET statistical report CON and ULTRA methods

	BET plot					Langmuir plot		
	V_m_	a_s, BET_	C	Total pore volume (*p*/*p*_0_ = 0.990)	Mean pore diameter	Vm	a_s, Lang_	B
	[cm^3^(STP) ·g^− 1^]	[m²·g⁻¹]		[cm³·g⁻¹]	[nm]	[cm^3^(STP) ·g^− 1^]	[m²·g⁻¹]	
CON Method	0.63	2.73	9.12	0.006	8.63	15.01	65.33	0.0020
ULTRA Method	0.32	1.40	14.46	0.005	14.70	7.52	32.72	0.0026

**Fig. 6:**
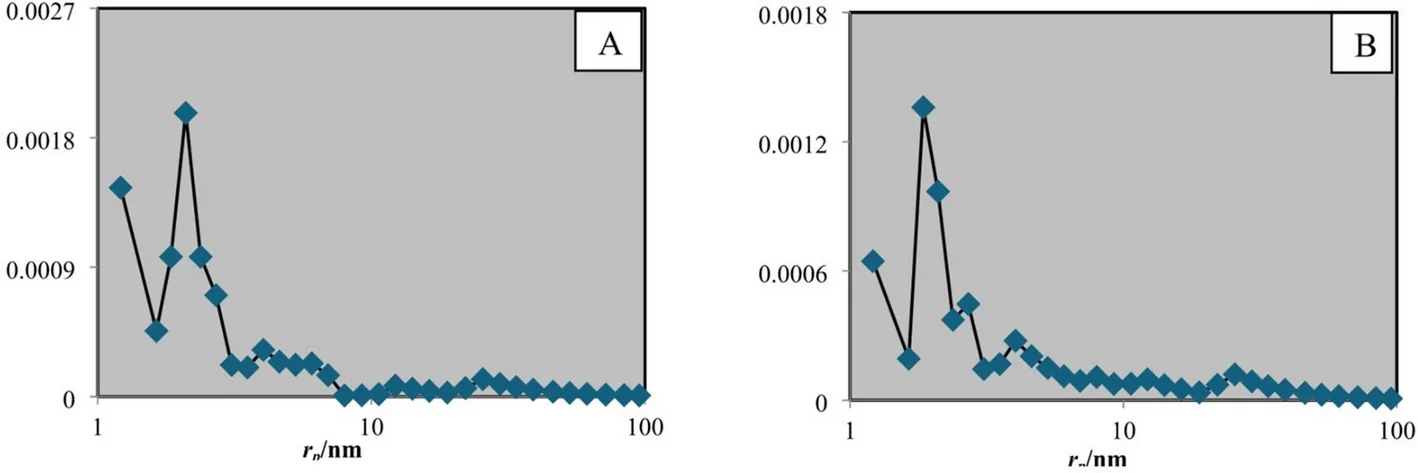
BJH plot of samples synthesized by (**a**) CON and (**b**) ULTRA methods

Adsorption–desorption isotherms and Barrett–Joyner–Halenda (BJH) method further confirmed the textural characteristics of the Ta-MOF samples. Both CON and ULTRA exhibited a broad pore size distribution from approximately 1.2 to 95 nm, with the highest pore volume observed in the microporous region and additional pores distributed across larger sizes. Adsorption gradually increased with relative pressure for both samples, consistent with the presence of accessible pore structures (Fig. 6). The CON sample exhibited slightly higher adsorption capacity, consistent with its higher BET surface area, while the ULTRA sample showed larger average pore diameters. Overall, the results demonstrate that both synthesis methods produced Ta-MOF materials with comparable textural properties, which likely facilitate metal ion diffusion and provide accessible adsorption sites within the framework.

The adsorption efficiency for As, Mo, and Cd is closely related to these structural features. The CON sample, with a slightly higher surface area and pore volume, provides more active sites, and the mesoporous network facilitates ion access in both samples, enhancing overall adsorption. These results highlight the importance of synthesis methods in optimizing MOF structure for effective heavy metal removal.

### Statistical analysis

The experimental data were analyzed using Design Expert software, a widely accepted platform for response surface methodology (RSM) and multivariate optimization. A second-order polynomial model was employed to describe the relationship between the operating variables and heavy metal removal efficiency, as it effectively captures both nonlinear effects and interaction behavior among parameters.

The model incorporated linear terms for pH (A), initial metal concentration (B), adsorbent dosage (C), and contact time (D), along with interaction terms (AB, AC, AD, BC, BD, CD) and quadratic terms (A², B², C², D²) to account for curvature effects in the response surface. Model simplification was performed through statistical significance testing, where terms with p-values below 0.05 were considered significant contributors, while marginal terms (0.05–0.1) were retained when they showed potential influence on system behavior. Insignificant terms (*p* > 0.1) were removed to improve model clarity and robustness, resulting in a refined predictive equation for heavy metal removal [40].

The statistical significance and adequacy of the model were assessed using analysis of variance (ANOVA), where F-values and p-values were used to determine the contribution of each term. The second-order structure allowed accurate representation of both main effects and interaction effects, improving the reliability of predictions across the experimental domain. This statistical refinement ensured strong agreement between experimental and predicted values, confirming the robustness of the developed model [41].

The detailed ANOVA results are presented in Table 3. The model p-value of less than 0.0001 confirms its high statistical significance, indicating that the observed variations in removal efficiency are not due to random error. In addition, the lack-of-fit p-values of 0.122 for molybdenum, 0.086 for arsenic, and 0.627 for cadmium indicate that the lack of fit is not statistically significant, confirming that the model adequately describes the experimental data and is suitable for prediction and optimization of heavy metal adsorption performance.

**Table 3:** ANOVA results for the removal percentages of Mo, As, and Cd ions

Mo						
Source	Sum of squares	df	Mean square	F-value	*p*-value	
Model	4025.48	8	503.19	70.43	< 0.0001	significant
A-pH	1083.94	1	1083.94	151.71	< 0.0001	
B-Conc	217.74	1	217.74	30.48	< 0.0001	
C-Aasorb	337.58	1	337.58	47.25	< 0.0001	
D-Time	896.83	1	896.83	125.52	< 0.0001	
A²	1235.98	1	1235.98	172.99	< 0.0001	
B²	42.54	1	42.54	5.95	0.0236	
C²	23.93	1	23.93	3.35	0.0815	
D²	425.86	1	425.86	59.60	< 0.0001	
Residual	150.04	21	7.14			
Lack of Fit	135.42	16	8.46	2.89	0.1223	not significant
Pure Error	14.62	5	2.92			
Cor Total	4175.53	29				

**As**.						
Source	Sum of squares	df	Mean square	F-value	*p*-value	
Model	8895.16	7	1270.74	30.77	< 0.0001	significant
A-pH	1719.92	1	1719.92	41.64	< 0.0001	
B-Conc	1899.44	1	1899.44	45.99	< 0.0001	
C-Adsorb	1270.94	1	1270.94	30.77	< 0.0001	
D-Time	1610.65	1	1610.65	39.00	< 0.0001	
A²	2206.28	1	2206.28	53.42	< 0.0001	
C²	256.29	1	256.29	6.21	0.0208	
D²	256.29	1	256.29	6.21	0.0208	
Residual	908.61	22	41.30			
Lack of Fit	837.87	17	49.29	3.48	0.0863	not significant
Pure Error	70.74	5	14.15			
Cor Total	9803.77	29				

Cd.						
Source	Sum of squares	df	Mean square	F-value	*p*-value	
Model	6730.89	6	1121.82	64.31	< 0.0001	significant
A-pH	1609.66	1	1609.66	92.28	< 0.0001	
B-Conc	1176.98	1	1176.98	67.47	< 0.0001	
C-Adsorb	623.12	1	623.12	35.72	< 0.0001	
D-Time	692.19	1	692.19	39.68	< 0.0001	
A²	2575.77	1	2575.77	147.66	< 0.0001	
D²	166.03	1	166.03	9.52	0.0052	
Residual	401.20	23	17.44			
Lack of Fit	304.36	18	16.91	0.8731	0.6270	not significant
Pure Error	96.84	5	19.37			
Cor Total	7132.09	29				

After excluding non-significant variables, a final response surface model was developed to optimize heavy metal adsorption (Table 4). The model is based on the most influential parameters and shows strong statistical consistency, indicating reliable predictive capability across the studied system. The coefficient of determination (R²) values confirm a high level of agreement between experimental and predicted responses for all three metals. The R^2^ values are 0.9073 for arsenic, 0.9738 for molybdenum, and 0.9437 for cadmium, demonstrating that the model explains a large portion of the observed variability and provides a strong fit to the experimental data. Model adequacy was further evaluated using Adequate Precision, which compares the signal-to-noise ratio of the model. The obtained values of 21.19 for arsenic, 37.25 for molybdenum, and 33.92 for cadmium are well above the acceptable threshold of 4, confirming a sufficient signal for reliable prediction within the design space. The Adjusted R² values (0.87 for arsenic, 0.96 for molybdenum, and 0.92 for cadmium) indicate good agreement between model complexity and data representation, while the Predicted R² values (0.73 for arsenic, 0.91 for molybdenum, and 0.85 for cadmium) confirm satisfactory predictive performance, particularly for molybdenum and cadmium. The close relationship between adjusted and predicted values further supports model stability and robustness.

Overall, the statistical indicators collectively confirm that the developed RSM model is reliable, well-fitted, and capable of accurately describing and predicting heavy metal adsorption behavior within the investigated experimental range. 

**Table 4:** Statistical data on the removal percentages of As, Mo, and Cd ions

	As	Mo	Cd
*R* ^2^	0.9073	0.9738	0.9437
Adjusted R^2^	0.8778	0.9638	0.9291
Predicted R^2^	0.7362	0.9158	0.8566
Adeq Precision	21.1956	37.2543	33.9283

**Fig. 7:**
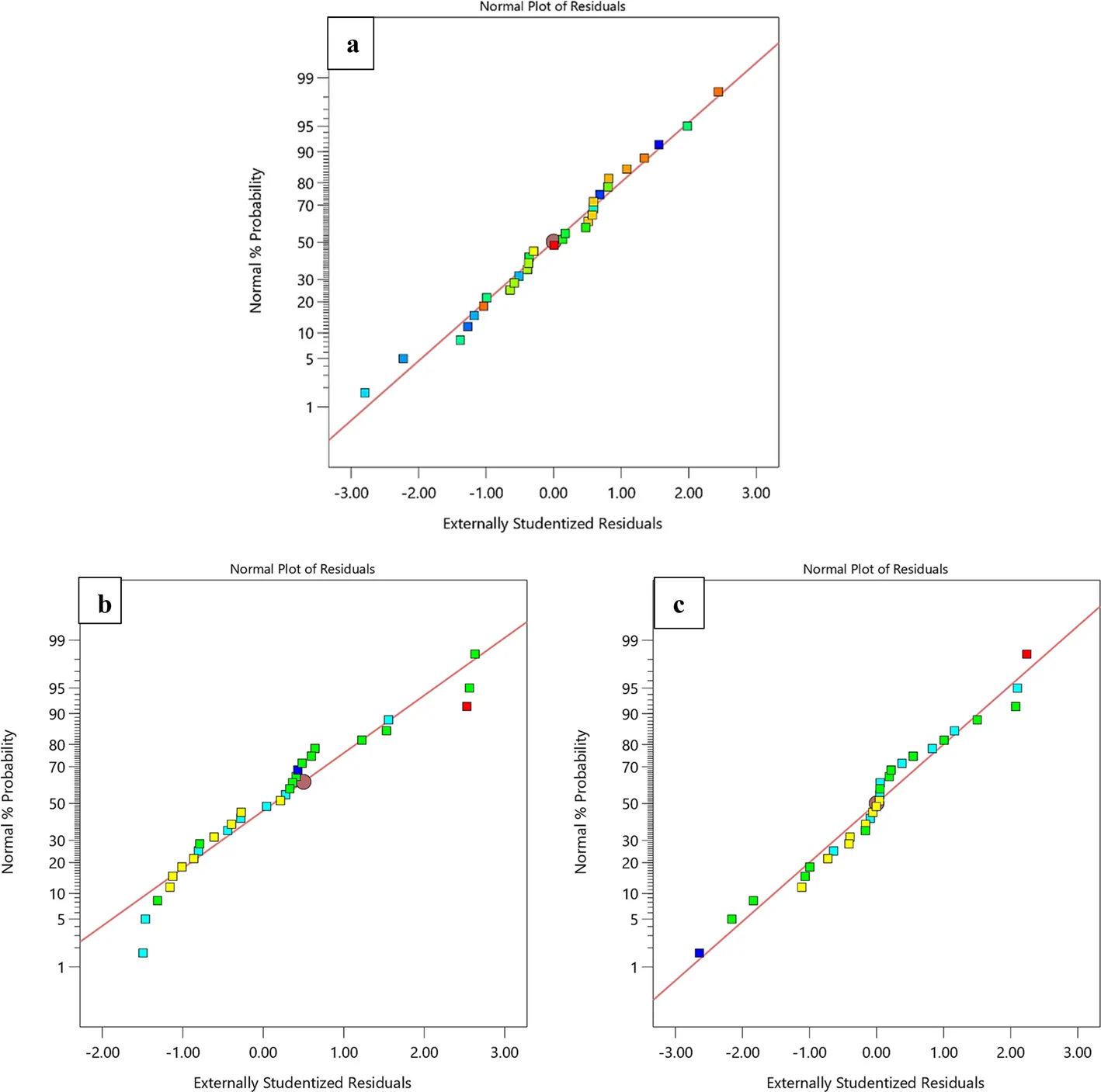
Normal probability plot of residuals for the removal of (**a**) As, (**b**) Mo, and (**c**) Cd

To evaluate the reliability of the model and experimental data, the normal probability plot of residuals (Fig. 7) was analyzed. The residuals are closely aligned along the straight reference line, indicating that the error distribution follows a near-normal pattern without significant deviations or abnormal trends.

This linear alignment confirms that the model errors are randomly distributed and that no systematic bias or hidden variables significantly affect the predictions. The absence of noticeable curvature or scattered deviations further supports the adequacy of the model fit and the reliability of the regression results. Overall, the residual analysis verifies that the proposed model is statistically valid, well-fitted to the experimental data, and suitable for accurate prediction within the studied range.

#### Evaluation of model quality via predicted values

**Fig. 8:**
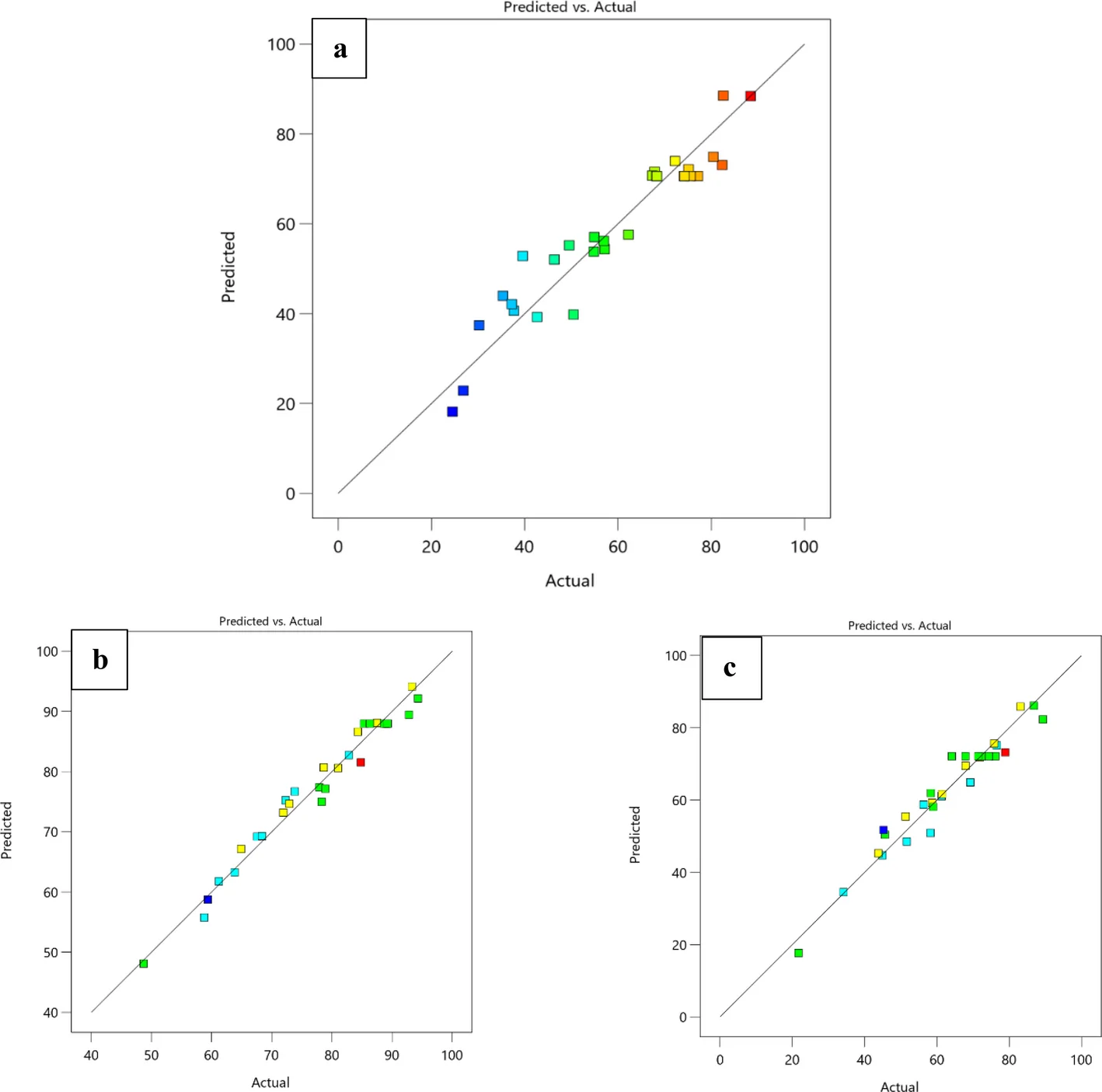
Predicted vs. actual values for the removal efficiency of (**a**) As, (**b**) Mo, and (**c**) Cd

The quality of the proposed model was further evaluated by comparing the predicted values directly with the corresponding experimental results. In a reliable model, the predicted data should align closely with the 45° reference line, indicating strong agreement between simulation and experiment. As shown in Fig. 8, the predicted values are uniformly distributed and closely clustered along this diagonal, demonstrating that the model accurately reproduces the experimental observations. This strong alignment confirms that the model is neither overfitted nor biased and that it successfully captures the true behavior of the system, enabling confident prediction of new data.

**Fig. 9:**
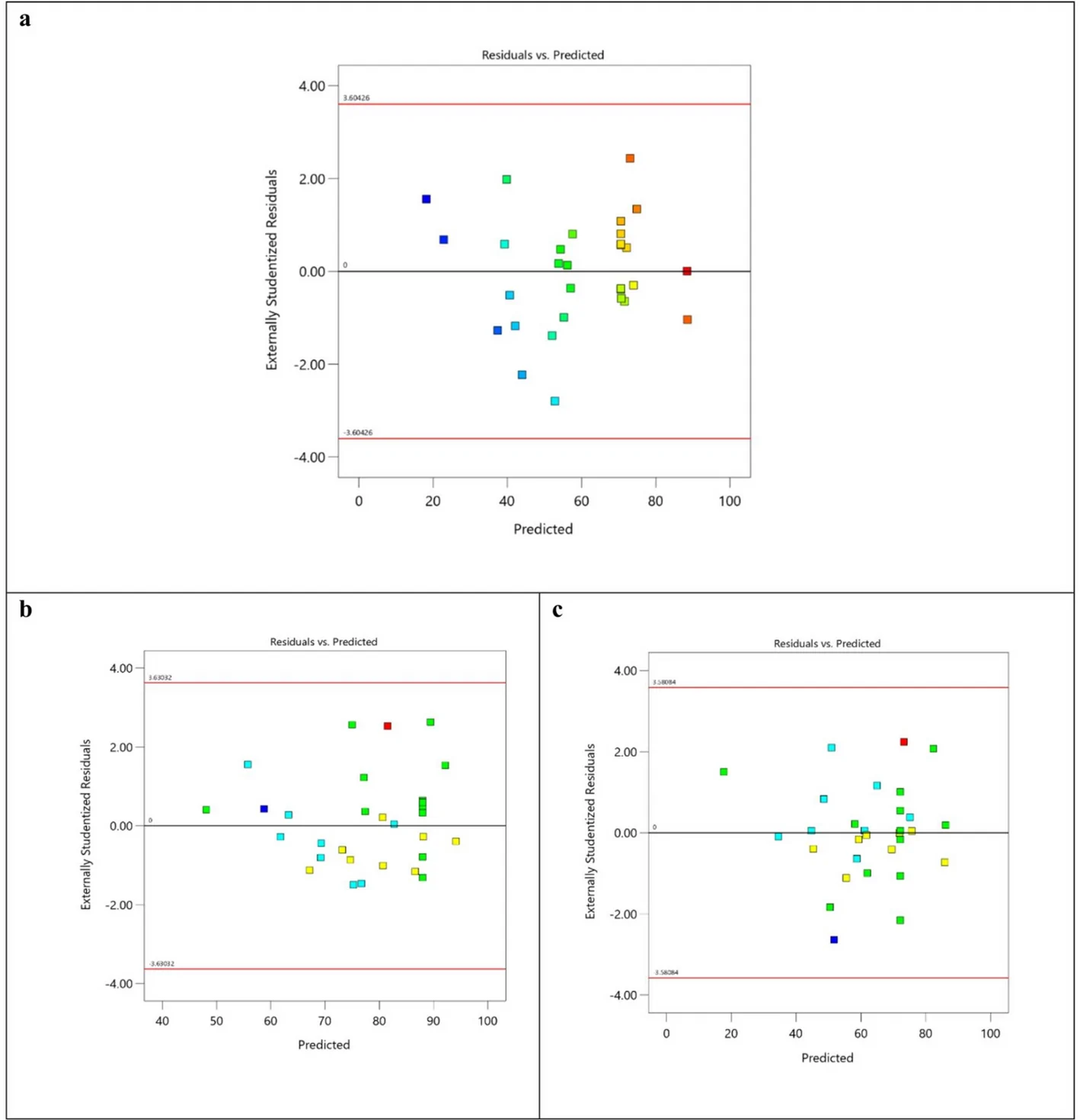
Residuals vs. predicted values for the removal efficiency of (**a**) As, (**b**) Mo, and (**c**) Cd

Model robustness was additionally assessed through analysis of the prediction errors, presented in Fig. 9. In this plot, predicted values are ordered in ascending sequence and their associated errors are examined. A well-calibrated model is characterized by randomly distributed errors without systematic trends and with magnitudes confined within acceptable statistical limits. In the present case, all prediction errors remain within ± 3.6 standard deviations and exhibit no discernible pattern, indicating the absence of structural bias or instability. Collectively, these results confirm that the model is statistically sound, stable, and suitable not only for fitting the current experimental data but also for reliable prediction and future validation studies.

#### Heavy metal adsorption optimization

To optimize the removal of cadmium, molybdenum, and arsenic, this study applied Response Surface Methodology (RSM) to systematically evaluate all operational variables within defined ranges. The model predicted removal efficiencies of 89.76% for arsenic, 88.94% for cadmium, and 94.32% for molybdenum, closely matching the experimental values of 87.63%, 89.21%, and 94.19%, respectively (Table 5). This strong agreement confirms that the model accurately captures the system’s behavior, with only minor deviations likely due to measurement precision or slight experimental variations. The close correspondence between predicted and observed results demonstrates the model’s reliability for optimizing heavy metal removal and suggests it can predict system performance under new or untested conditions.

**Table 5:** Optimum conditions for the removal of As Cd, Mo

Pollutant	pH	Initial concentration	Adsorbent dosage	Time	Predicted Removal	Actual Removal
Arsenic	6.97	33.32	37.64	43.96	89.76	87.63
Cadmium	6.64	32.50	40	46.25	88.94	89.21
Molybdenum	6.67	33.15	34.96	39.77	94.32	94.19

#### Variable effects

**Fig. 10:**
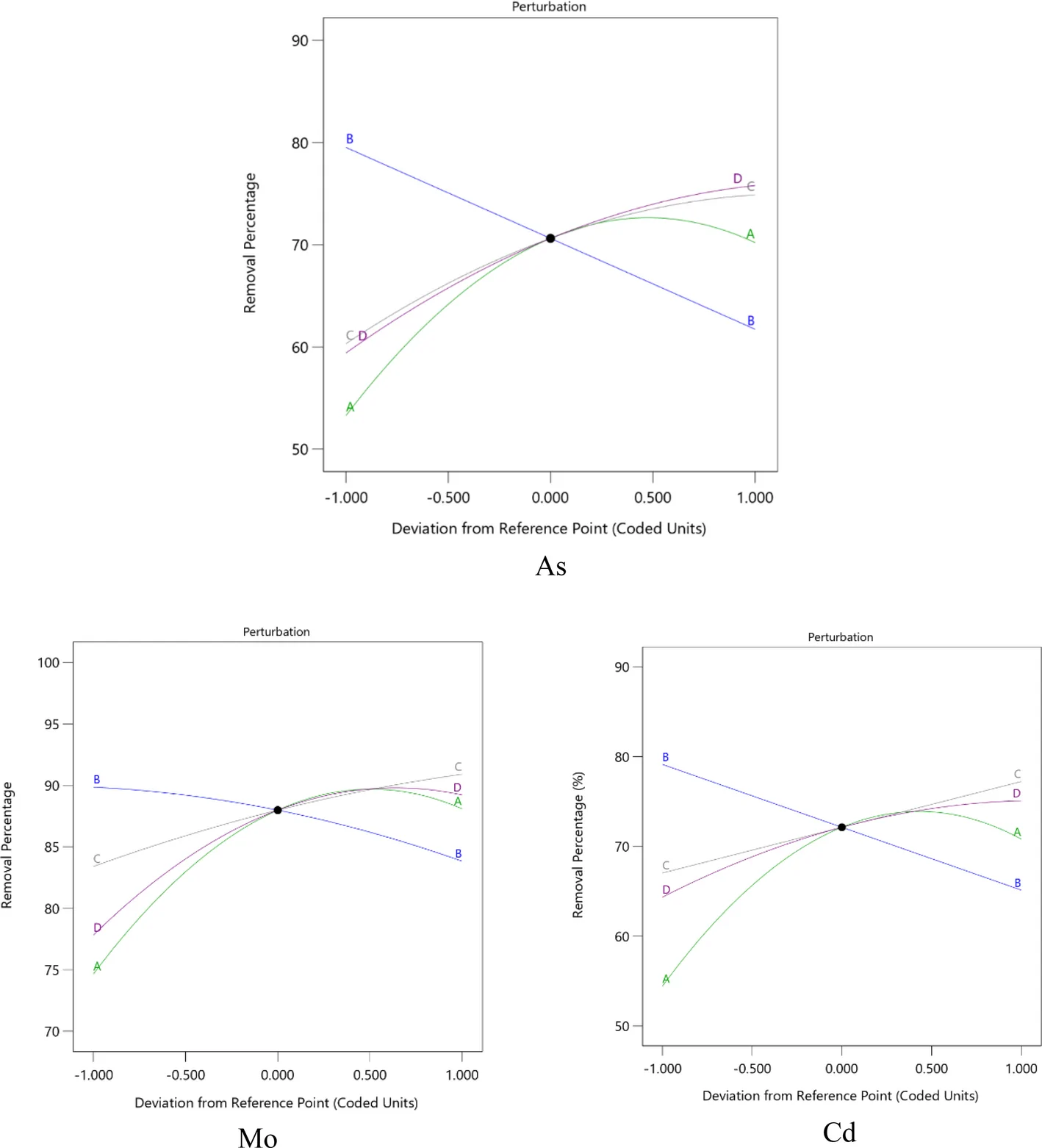
Perturbation plots for As,Mo and Cd (A:pH, B:initial concentration, C:adsorbent dosage and D:time)

To thoroughly understand the factors affecting heavy metal removal, the influence of key variables was analyzed using perturbation charts from Design Expert software. These charts allow each parameter to be evaluated individually while keeping others constant, clearly showing that although all variables affect the process, they do so at different rates (Fig. 10). The effects of initial concentration and contact time are further detailed in the isotherm and kinetic sections.

Increasing the adsorbent dosage consistently improved the removal of arsenic, molybdenum, and cadmium, mainly because more active sites become available on the Ta-MOF surface. With higher dosage, there are more binding locations for the ions, which reduces competition and allows more effective uptake until the system gradually approaches a near-saturation state where additional sites contribute less to overall improvement. The effect of pH (3–9) showed a clear and non-uniform influence on all three metals, confirming it as the key RSM controlling factor. For arsenic, removal increased from 60.43% at pH 3.12 to 75.68% at pH 7.52 and then remained relatively stable under near-neutral conditions. This behavior is linked to reduced proton competition at higher pH and more favorable interaction between deprotonated Ta-MOF surface sites and arsenic species. This is likely mediated by ligand-exchange (inner-sphere complexation) with arsenate rather than simple electrostatic attraction, consistent with the coordination-based mechanism discussed in Sect. 3.6. For molybdenum, removal rose from 24.31% at pH 3.08 to a maximum of 81.47% at pH 6.58, showing that mildly acidic to near-neutral conditions are most favorable. After this point, efficiency decreased under alkaline conditions, most likely due to changes in molybdenum species in solution that reduce their affinity for the adsorbent surface. Cadmium showed a similar trend, increasing from 21.54% at pH 3.15 to 80.62% at pH 6.66, then decreasing at higher pH values. At low pH, strong competition from H⁺ ions limits adsorption, while at higher pH, changes in surface charge balance and ion interaction reduce uptake efficiency. The observed pH dependence suggests that changes in surface charge, proton competition, and the aqueous speciation of the adsorbates contribute to the adsorption behavior. Overall, the different pH profiles observed for Cd, As, and Mo reflect the combined effects of Ta-MOF surface chemistry and the ionic forms of the species present in solution. This highlights pH as an important factor controlling the adsorption process.

### Kinetic analysis of adsorption

**Fig. 11:**
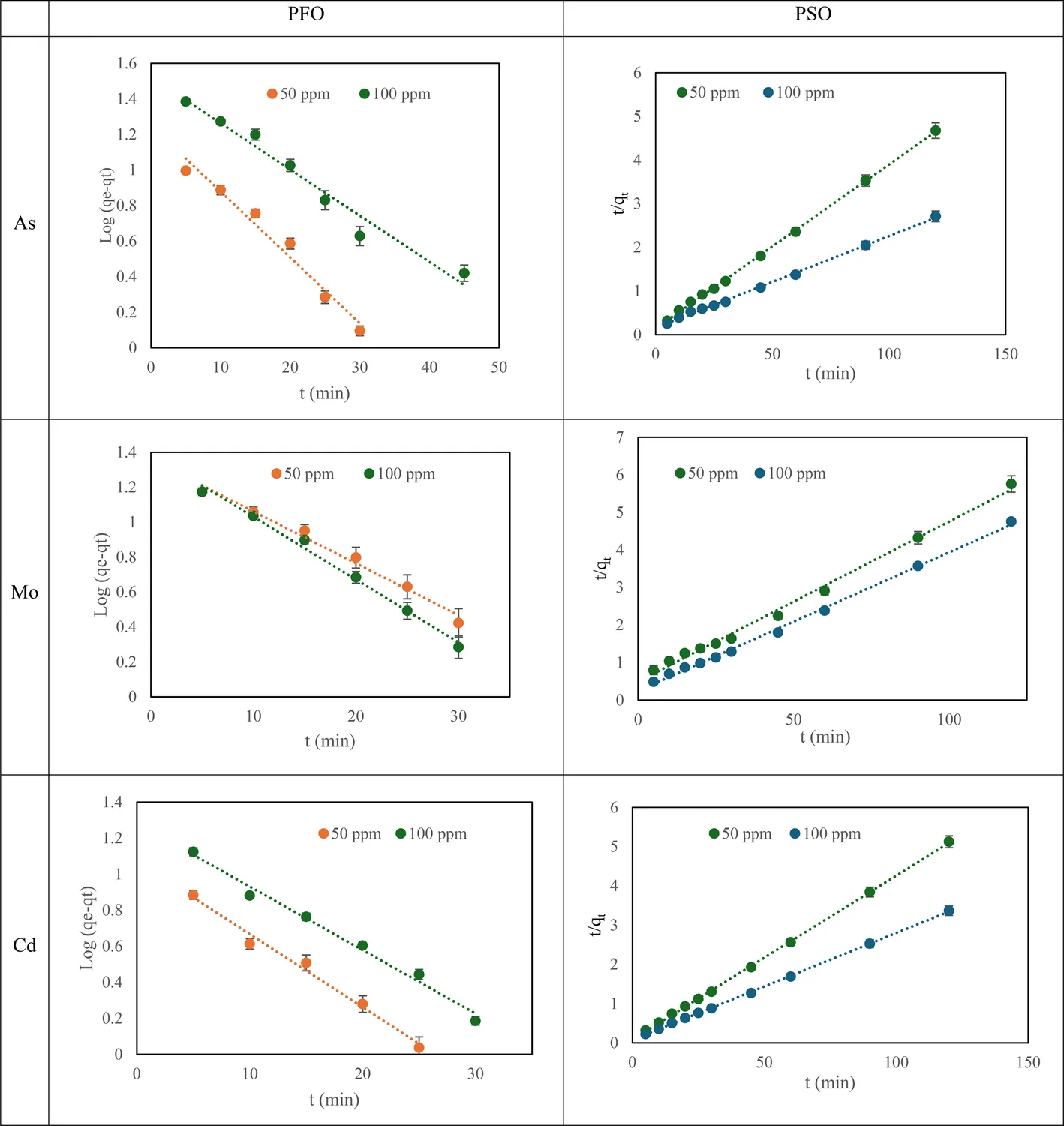
Pseudo First Order and Second Order Kinetics plot at different initial concentrations for As, Mo and Cd adsorption

The duration of contact between the adsorbent and the contaminant plays a pivotal role in the design and optimization of adsorption-based remediation systems, directly influencing the efficiency and kinetics of pollutant removal. In order to investigate the time-dependent behavior of the synthesized Ta-MOF, the influence of contact time on the adsorption capacity was systematically examined under controlled experimental conditions, with pH values maintained at their respective previously optimized levels. Kinetic experiments were performed at two different initial concentrations 50 mg·L^− 1^ and 100 mg·L^− 1^ for each of the target heavy metal ions (As, Mo, and Cd), allowing for comparative evaluation across varying contaminant loads. The experimental data obtained from these studies were subjected to kinetic modeling using both the pseudo-first-order (PFO) and pseudo-second-order (PSO) equations, as represented by Eqs. 3  and 4, respectively, to determine the underlying adsorption mechanisms and rate-controlling steps. All kinetic measurements were conducted in triplicate to ensure reproducibility and statistical reliability. The results are reported as the mean values derived from these independent runs, and half-range error bars have been calculated and included to represent the variability within each data set. This methodological rigor supports the robustness of the kinetic evaluation and enhances the confidence in the observed adsorption trends and model fitting. The graphical representations of t/q_t_ and Log (q_e_-q_t_) versus time (t) are illustrated in Fig. 11, and also kinetic parameters are summarized in Table 6.

**Table 6:** Kinetic parameters for the adsorption of As, Mo, and Cd ions on Tantalum MOF

		Pseudo-1st -order				Pseudo-2nd -order			
	C_i_ (ppm)	k_1_ (min^− 1^)	q_(e, Exp)_* (mg·g⁻¹)	q_(e, Cal)_* (mg·g⁻¹)	R^2^	k_2_ (g·mg^− 1^·min^− 1^)	q_(e, Exp)_ (mg·g⁻¹)	q_(e, Cal)_ (mg·g⁻¹)	R^2^
As	50	0.08567	25.66	17.91843	0.969	0.00997	25.66	26.59574	0.9994
	100	0.06011	44.26	33.51197	0.968	0.002814	44.26	47.61905	0.9983
Mo	50	0.069	20.84	22.58	0.978	0.003705	20.84	23.47418	0.9945
	100	0.0826	25.23	24.53	0.992	0.005555	25.23	27.10027	0.9975
Cd	50	0.07531	23.42	11.87	0.97	0.020556	23.42	23.92344	0.9997
	100	0.09419	35.645	19.27	0.935	0.009559	35.645	36.76471	0.9997

The adsorption behavior of arsenic, molybdenum, and cadmium is well described by the pseudo-second-order kinetic model, while the pseudo-first-order model shows comparatively poor fitting based on regression analysis. The high correlation coefficients (R^2^ > 0.99) obtained for the pseudo-second-order model indicate a good agreement between experimental and calculated values over the entire adsorption time range. In addition, the close correspondence between calculated and experimental adsorption capacities (q_e_,_cal_ and q_e_,_exp_) suggests that the adsorption process is strongly dependent on available active sites and surface interaction dynamics. Overall, the good fit to the pseudo-second-order model suggests that the adsorption process is likely surface-interaction-controlled, and chemical interactions may contribute to the uptake of Cd, As, and Mo alongside possible electrostatic attraction and coordination with functional groups on the MOF surface, although kinetic fitting alone cannot conclusively establish the nature of these interactions. This behavior is consistent with the favorable adsorption performance observed in the equilibrium studies [42].

Under near-neutral pH conditions, Cd(II) is predominantly present as a cation, whereas arsenate and molybdate species are mainly present as oxyanions. Therefore, their interactions with the Ta-MOF surface may not be identical and could involve different combinations of electrostatic attraction, surface complexation, and functional-group affinity. Further spectroscopic investigations are required to distinguish the specific adsorption pathways of each species.

### Adsorption isotherm study

**Fig. 12:**
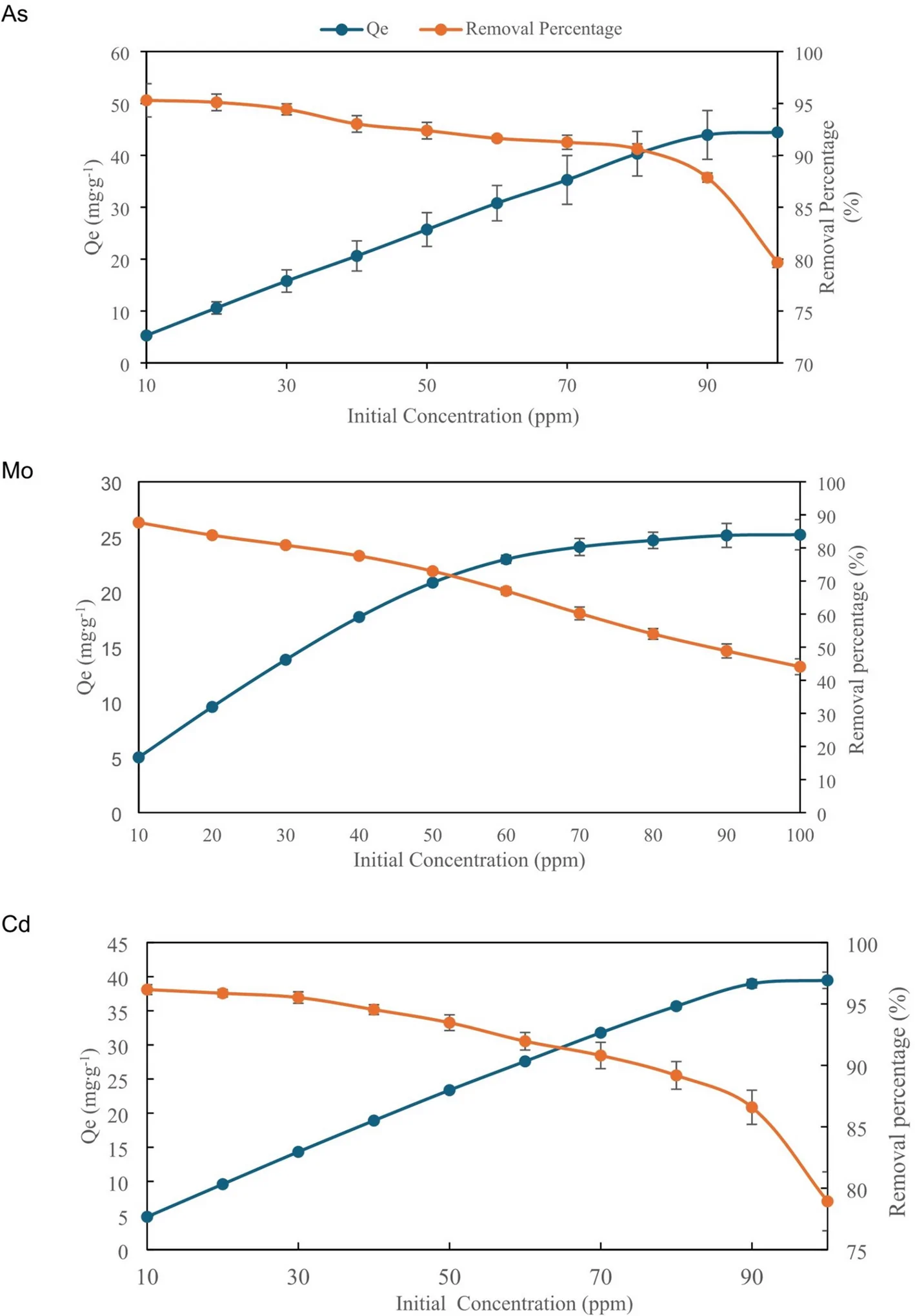
Removal percentage and adsorption capacity of removal process of As, Mo, and Cd

The relationship between adsorption capacity and the initial heavy metal concentrations within the investigated range is illustrated in Fig. 12. The results indicate that the maximum adsorption capacities were found to be 44.26 mg·g⁻¹ for arsenic, 25.19 mg·g⁻¹ for molybdenum, and 39.46 mg·g⁻¹ for cadmium at concentrations of 100 mg·L^− 1^ for all of the pollutants. As the concentrations of As, Mo, and Cd ions increased, a notable increase in adsorption capacities was observed, and this trend continued until a plateau was reached, as depicted in Fig. 12. Additionally, the findings demonstrate that as the concentration of the adsorbate rises, the removal efficiency declines, with values decreasing from 95.3% to 79.69% for arsenic, from 87.68% to 44.09% for molybdenum, and from 96.2% to 78.93% for cadmium. The decrease in adsorption performance at higher pollutant concentrations is mainly due to the limited number of available active sites on the surface of the adsorbent. When the initial concentration of pollutants is low, more adsorption sites are empty. This creates a stronger concentration gradient between the solution and the adsorbent, which helps the pollutants move quickly and easily toward the material. As a result, the contact between the pollutants and the Ta-MOF is better, which increases removal efficiency. However, when the concentration becomes higher, too many pollutant molecules compete for fewer sites, and this reduces the overall adsorption rate.

These findings are consistent with the classical adsorption theory and are further substantiated by data obtained through isotherm studies conducted in triplicate to ensure reproducibility. The consistency of results across independent experimental runs affirms the statistical reliability and experimental validity of the observed trends. Furthermore, the inclusion of half-range error bars supports the robustness and precision of the measurements, confirming that the decline in performance at elevated concentrations is a reproducible and statistically significant phenomenon.

The characterization of adsorption isotherms is important for the design and evaluation of adsorption systems and for understanding the equilibrium behavior between adsorbate species and the adsorbent surface. These models provide a framework to describe how metal ions distribute between the liquid phase and the solid phase at equilibrium. Linear regression analysis is commonly applied to evaluate the suitability of isotherm models and to determine how well they represent the experimental data.

In this study, equilibrium experiments were conducted to determine the adsorption capacity of the Tantalum MOF for arsenic, molybdenum, and cadmium ions. A range of initial pollutant concentrations was applied under controlled pH conditions specific to each metal ion. A previously optimized adsorbent dosage was used, and the contact time was maintained at 1 h to ensure equilibrium conditions. The experimental data obtained from batch adsorption tests were analyzed using Langmuir, Freundlich, and Temkin isotherm models.

The Langmuir adsorption model, a fundamental aspect of equilibrium isotherms, provides a robust framework for quantifying and comparing the adsorption capacities of different adsorbents. It is based on the assumption that adsorption occurs solely at homogeneous sites on the solid matrix, leading to the formation of monolayers without significant intermolecular interactions. This model provides a theoretical framework for understanding surface coverage by equating the opposing rates of adsorption and desorption, ultimately leading to the establishment of a dynamic equilibrium state. The Langmuir isotherm equation, in its linearized form, incorporates critical parameters such as the equilibrium concentration of pollutants C_e_, maximum adsorption capacity q_max_, and the Langmuir constant K_L_, which indicates the affinity between the adsorbate and the adsorbent. Particularly noteworthy is the dimensionless separation factor R_L_ intrinsic to the Langmuir isotherm, which serves as a vital indicator of the nature of the adsorption process. R_L_ values greater than 1 suggest an unfavorable adsorption process, while values between 0 and 1 indicate favorable adsorption, with R_L_ = 1 signifying linear adsorption behavior and R_L_=0 indicating irreversible adsorption.

**Fig. 13:**
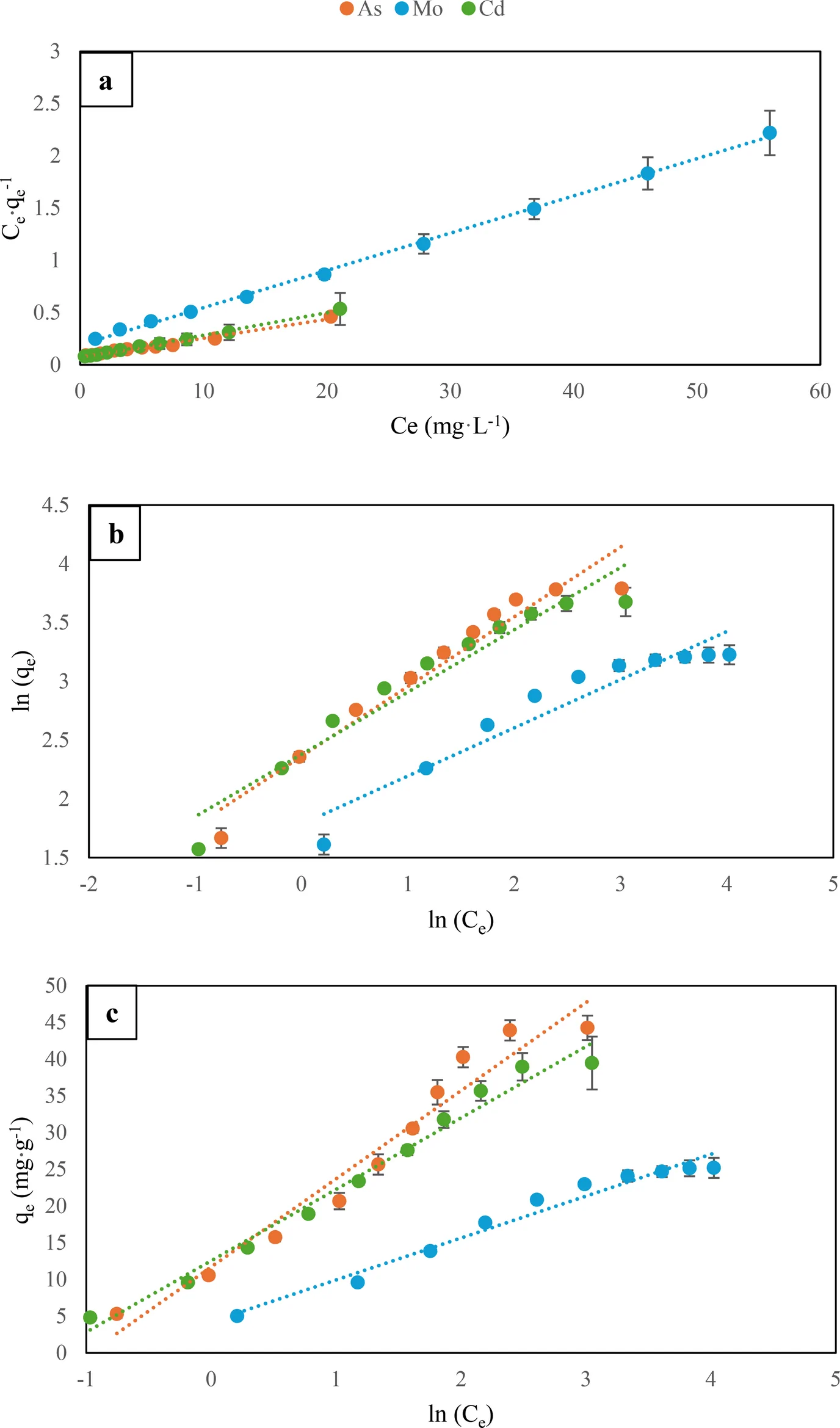
Adsorption isotherms for removal of As, Mo and Cd using (**a**) Langmuir, (**b**) Freundlich and (**c**) Temkin

Insights derived from the Langmuir isotherm analysis are invaluable for optimizing adsorption processes and designing effective adsorption systems tailored to the specific removal requirements of pollutants. The maximum adsorption capacities (q_max_), as determined by Langmuir isotherm analysis (refer to Fig. 13; Table 7), were found to be 55.24 mg·g⁻¹ for arsenic, 28.01 mg·g⁻¹ for molybdenum, and 46.29 mg·g⁻¹ for cadmium ions, respectively. Remarkably, the adsorption process exhibited high efficacy for these pollutants. The Langmuir isotherm provided a good fit to the experimental adsorption data, with correlation coefficients of R^2^ ranging from 0.984 to 0.999 (Table 7). This result indicates that the adsorption process can be reasonably described by monolayer adsorption on relatively homogeneous adsorption sites. The high calculated q_max_ values further demonstrate the high adsorption capacity of the Ta-MOF toward the investigated metal ions. In addition, the calculated (R_L_) values were within the favorable range of 0–1, confirming the favorable nature of the adsorption process. However, the Langmuir constants alone cannot be used to determine the strength or specific chemical nature of the interactions between the metal ions and the Ta-MOF. The adsorption mechanism is therefore discussed based on the combined evidence from the isotherm and kinetic models, FTIR analysis, and textural characteristics of the material. It should be noted that q_max_ and K_L_ are mathematically independent parameters within the Langmuir framework: q_max_ reflects the estimated maximum adsorption capacity associated with the available adsorption sites, whereas K_L_ is related to the affinity of the adsorbate for these sites. These parameters therefore describe different aspects of the adsorption behavior, and a high q_max_ value does not require a correspondingly high K_L_, and the two parameters should not be interpreted as contradictory. Moreover, the calculated R_L_ values (0.01–0.58) fall well within the favorable range (0 < R_L_ < 1), indicating favorable adsorption under the experimental conditions. The high Langmuir fitting quality (R^2^ = 0.984–0.999), together with the high q_max_ and favorable R_L_ values, supports the applicability of the Langmuir model under the experimental conditions.

Beyond the Langmuir model, the equilibrium adsorption data were further analyzed using the Freundlich isotherm, which describes adsorption on a heterogeneous surface featuring a non-uniform distribution of active sites and associated adsorption energies, typically expressed through an exponential relationship. Essentially, the Freundlich isotherm model emphasizes the non-uniform, multilayer, and heterogeneous nature of the adsorption process. Within the framework of the Freundlich isotherm model, the parameters 1/n_F_ and K_f_ signify the adsorption intensity and the Freundlich empirical constant related to the adsorption capacity of the adsorbate, respectively. The Freundlich constant K_f_ is related to adsorption capacity, whereas n_F_ (or 1/n_F_) reflects adsorption intensity and surface heterogeneity. Favorable adsorption conditions are typically characterized by 0 < 1/ n_F_ < 1. As detailed in Table 7, the 1/ n_F_ values for the evaluated pollutants were less than unity, indicating favorable adsorption on a heterogeneous surface [43]. However, the Freundlich model does not by itself provide definitive evidence regarding the nature of the adsorption mechanism.

Additionally, the Temkin isotherm model displayed compatibility with the adsorption system involving the examined adsorbent, as evidenced by the linear curve presented (refer to Fig. 13). The adsorption behavior of the target pollutants was satisfactorily interpreted using the Temkin isotherm model, as indicated by a reasonably strong correlation coefficient. However, the overall analysis suggests that the adsorption of arsenic, molybdenum, and cadmium ions onto the Tantalum-based metal-organic framework is most accurately characterized by the Langmuir isotherm model, with the Temkin model providing a secondary but supportive fit.

Overall, the adsorption of Cd, As, and Mo onto Ta-MOF likely proceeds through several concurrent interactions rather than a single dominant mechanism. The FTIR spectra of the as-synthesized Ta-MOF (Sect. 3.1) identified carboxylate (1400 and 1600–1700 cm^− 1^) and pyridinic-nitrogen groups originating from the ligand backbone, which can act as Lewis base donor sites and may provide potential coordination sites for metal cations such as Cd(II), while certain Ta centers within the framework may also act as Lewis acid sites, potentially interacting with oxyanionic species such as arsenate and molybdate through surface complexation. Consistent with the pH-dependent speciation discussed above, deprotonation of surface carboxylate and hydroxyl groups under the near-neutral operating conditions of this study may favor a more negatively charged surface, which could promote electrostatic attraction of the Cd(II) cation, while the oxyanionic As and Mo species may interact with these Ta sites through surface complexation, in addition to possible electrostatic contributions. In addition, the broad pore-size distribution obtained from BJH analysis (approximately 1.2–95 nm) suggests that steric exclusion is unlikely to be a major limitation for ion access and may facilitate diffusion of dissolved species toward internal binding sites, consistent with the rapid attainment of adsorption equilibrium observed experimentally. This proposed mechanism should be regarded as a preliminary interpretation, and further spectroscopic studies would help verify it in future work. It is, however, consistent with the isotherm, kinetic, pH, FTIR, and textural characterization data obtained in this work, and offers a reasonable explanation for the high removal efficiencies and favorable isotherm fits observed for all three pollutants.

**Table 7:** Isotherm constants and parameters for the adsorption of As, Mo, and Cd ions on Tantalum MOF

Isotherm model	Parameters	Linearized isotherm		
		As	Mo	Cd
Langmuir	q_Max_. (mg·g⁻¹)	55.24	28.01	46.29
	K_L_ (L·mg^− 1^)	0.25	0.187	0.328
	R_L_	0.046–0.58	0.01–0.41	0.04–0.58
	R^2^	0.984	0.9988	0.996
Freundlich	1/n_F_	0.593	0.409	0.530
	K_F_ [(mg·g⁻¹)(L·mg^− 1^)^1/n^]	10.622	5.953	10.794
	R^2^	0.941	0.909	0.936
Temkin	β_T_	11.99	5.690	9.722
	K_T_ (L·g^− 1^)	2.654	2.108	3.638
	R^2^	0.956	0.969	0.981

Finally, Table 8 presents a comparative analysis of the results obtained in this study alongside those reported in previous studies. This comparison includes key parameters such as the type of adsorbent used, the targeted pollutant, and the corresponding adsorption capacity (mg·g⁻¹) reported in each study. The synthesized Ta-MOF exhibited adsorption capacities comparable to or higher than several previously reported adsorbents under the investigated experimental conditions.

**Table 8:** Comparison of the maximum adsorption capacity of Ta-MOF with previously reported adsorbents for Cd, As, and Mo removal

Adsorbent	Pollutant	Adsorption capacity (mg·g⁻¹)	References
Reduced Graphene Oxide/PANI/ZnO	Cd	22.522	[44]
metal-doped nickel ferrite		20	[43]
Biochar based on Wetland Emergent Plant Zizania caduciflora leaf		59.8	[45]
Brewed Tea leaf waste		2.468	[46]
TMU-5		43	[47]
NH_2_-functionalized Zr MOFs		43	[48]
MIL-100(Fe)		9	[49]
Magnetic Nanoparticles (Fe_3_O_4_) with surfactants		42.37	[50]
Tantalum-Based MOF		46.29	This study
Zr-Based MOF UiO 66-12-AA	As	35.5	[51]
Zr-Based MOF UiO 66-36-AA		40.2	
Fe-BTC		12.3	[52]
MOF-808		24.88	[53]
MIL-53(Fe)		21.3	[54]
ZIF-8		7.5	[55]
MIL-125(Ti)		46.34	[56]
Tantalum-Based MOF		55.24	This study
Alumina-embedded mesoporous silica	Mo	16.8	[57]
Mg-Fe-NO_3_-LDH		21.30	[58]
Zeolite-supported magnetite		18.02	[59]
Hydrotalcite-like layered double hydroxides		16.2	[60]
Tantalum-Based MOF		28.01	This study

The adsorption experiments in this study were performed using single-metal solutions under controlled laboratory conditions. In practical wastewater systems, multiple metal ions and other dissolved species may compete for available adsorption sites, potentially affecting adsorption efficiency and selectivity. Additionally, factors such as adsorbent regeneration and long-term operational stability can influence the practical applicability of Ta-MOF. While competitive adsorption and large-scale validation were not experimentally evaluated in the present study, previous research on MOF-based adsorbents suggests that adsorption selectivity depends on ionic charge, hydrated radius, and affinity to surface functional groups. These considerations indicate that the adsorption behavior of Ta-MOF in multicomponent and larger-scale systems may differ from that observed in single-metal batch experiments. Future work will focus on assessing adsorption selectivity, reproducibility, and performance under more realistic wastewater treatment conditions.

### Antibacterial assay

The antibacterial activity of the synthesized Ta-MOF was evaluated against two standard bacterial strains, Staphylococcus aureus and Escherichia coli, and the results are summarized in Table 9. The recorded values represent the mean inhibition zone diameters obtained from three independent replicates. The Ta-MOF exhibited greater antimicrobial activity against E. coli, with an average inhibition zone of 12.17 ± 0.15 mm, compared to S. aureus, which showed a slightly smaller inhibition zone of 10.47 ± 0.15 mm. The control group, using dimethyl sulfoxide (DMSO) as the solvent, showed no inhibition, confirming that the observed antibacterial activity originated from the Ta-MOF material.

The antibacterial activity of Ta-MOF may be attributed to several mechanisms commonly reported for metal-organic framework materials. Owing to its porous structure and the presence of abundant active surface sites, the material can establish close contact with bacterial cells, promoting surface interactions that may alter membrane permeability and affect normal cellular functions. The porous framework and the availability of surface-active sites may increase the probability of contact between bacterial cells and the adsorbent surface.

In addition, electrostatic interactions between the Ta-MOF surface and negatively charged bacterial cell membranes may contribute to bacterial inhibition. Such interactions can induce structural disturbances in the cell envelope, leading to changes in membrane integrity and transport processes. Previous studies have also suggested that metal-containing MOFs may promote the formation of reactive oxygen species (ROS), which can generate oxidative stress and damage essential cellular components, including proteins, lipids, and nucleic acids. Furthermore, the adsorption of biologically important nutrients and ions onto the MOF surface may reduce their availability to bacterial cells, thereby inhibiting microbial growth and reproduction [61].

The slightly higher inhibition observed against E. coli compared with S. aureus may be related to differences in cell wall structure between Gram-negative and Gram-positive bacteria, which can influence the interaction between bacterial cells and the MOF surface. Although the exact antibacterial mechanism was not directly investigated in the present study, the observed inhibition zones demonstrate that the synthesized Ta-MOF possesses measurable antibacterial activity against both bacterial strains. These findings indicate that the material may offer additional benefits beyond heavy metal adsorption, supporting its potential use in multifunctional water treatment applications.

**Table 9:** Inhibition zone diameters (mm) of Ta-MOF against E. coli and S. aureus (mean ± SD, *n* = 3)

Bacterial strain	Trial 1 (mm)	Trial 2 (mm)	Trial 3 (mm)	Mean ± SD (mm)
*Escherichia coli* (PTCC 1330)	12.3	12	12.2	12.17 ± 0.15
*Staphylococcus aureus* (PTCC 1112)	10.5	10.6	10.3	10.47 ± 0.15

## Conclusions

This study successfully synthesized and characterized a tantalum-based metal–organic framework (Ta-MOF) using CON and ULTRA methods. Characterization by FTIR, SEM, XRD, and BET confirmed the successful formation of the framework and revealed differences in the structural and morphological properties of the synthesized materials. The ULTRA method promoted improved crystallinity, enhanced structural uniformity, and reduced particle agglomeration, while the CON sample exhibited a slightly higher BET surface area. The adsorption performance of the synthesized Ta-MOF was evaluated for the removal of cadmium, arsenic, and molybdenum ions from aqueous solutions. Process optimization using Response Surface Methodology (RSM) identified the optimum operating conditions in terms of pH, initial metal concentration, adsorbent dosage, and contact time, resulting in high removal efficiencies for all investigated metal ions. Isotherm analysis showed good agreement with the Langmuir model, indicating monolayer adsorption behavior on relatively homogeneous adsorption sites. Kinetic studies demonstrated that the adsorption process was well described by the pseudo-second-order model, suggesting that adsorption is strongly influenced by the availability of active sites and surface interaction processes. Based on the isotherm parameters, FTIR functional-group assignments, pH-dependent speciation, and pore-size characteristics, the results suggest a possible adsorption mechanism involving surface coordination interactions, speciation-dependent electrostatic attraction, and pore-assisted diffusion. Morphological analysis revealed that ULTRA produced a more dispersed and less agglomerated structure with improved surface accessibility, which contributed positively to adsorption performance. In addition to its adsorption capability, the synthesized Ta-MOF exhibited measurable antibacterial activity against both Escherichia coli and Staphylococcus aureus, highlighting its potential as a multifunctional material for water treatment applications. Overall, the results demonstrate that Ta-MOF is an effective material for the removal of heavy metal contaminants from aqueous systems while providing supplementary antibacterial activity. The findings contribute to the growing development of MOF-based materials for environmental remediation and provide a basis for future studies involving regeneration, reusability, and multi-component adsorption systems under more realistic operating conditions.

## Data Availability

The data that support the findings of this study are available from the corresponding author upon reasonable request.
